# Sex differences in global disability-adjusted life years due to ischemic stroke: findings from global burden of diseases study 2019

**DOI:** 10.1038/s41598-022-10198-9

**Published:** 2022-04-14

**Authors:** Miaomiao Cao, Bolin Li, Jie Rong, Qian Li, Chaofeng Sun

**Affiliations:** 1 Department of Cardiology, Institute of Cardiovascular Channelopathy, Key Laboratory of Molecular Cardiology, First Affiliated Hospital of Xi’an Jiaotong University, Xi’an, 710061 Shaanxi People’s Republic of China; 2 Department of Encephalopathy, Affiliated Hospital of Shaanxi University of Chinese Medicine, Xianyang, People’s Republic of China

**Keywords:** Diseases, Neurology, Risk factors, Epidemiology

## Abstract

To investigate the sex differences in disability-adjusted life years (DALYs) due to ischemic stroke (IS) by year, location and age. We extracted sex-specific data on DALYs number, age-standardized DALYs rate (ASDR) and all-age DALYs rate of IS by year, location and age from the Global Burden of Diseases study 2019. The estimated annual percentage changes (EAPC) were calculated to evaluate the temporal trend of ASDR. For both sexes, although the ASDR of IS slightly decreased from 1990 to 2019, there has been an 60.3% increase in DALYs number worldwide. Sex difference in DALYs number (men minus women) decreased from − 2.83 million in 1990 to 0.14 million in 2019, while the men to women’s ASDR ratio slightly increased from 1.10 in 1990 to 1.21 in 2019. The sex differences in IS DALYs showed remarkable regional variation. The largest sex differences in DALYs number and ASDR were in China and Vietnam. Middle-aged men had a higher IS DALYs than their age-matched counterparts. High systolic blood pressure accounted for the highest DALYs number in 2019, but the top three attributable risk factors that had the greatest sex differences were tobacco, dietary risk, and alcohol use. Sex differences in IS DALYs varied by year, location and age, mostly attributed to the disproportion of cardiovascular risk factors between sexes. Considering the population growth and aging, it is necessary to monitor the sex difference in IS DALYs in different populations and thus provide evidence for local administration to improve current preventive and management strategies of IS.

## Introduction

Ischemic stroke (IS) accounts for almost 70–80% of stroke cases and is a significant cause of mortality and disability in many countries^1,2^. Sex differences in IS have been well described in several clinical and epidemiological studies^3^. Several studies reported that women had higher mortality of IS, a worse functional outcome, and quality of life for IS survivors than men. The sex differences in IS mortality disappeared after adjusting for confounders such as age and risk factors, but persisted for functional outcome and quality-of-life^4–6^. However, a meta-analysis including 19,652 patients showed that women with IS had better survival, but the worse quality of life and more disabilities than men^7^. Studies have provided a few possible explanations regarding sex disparities, mainly in the aspects of risk profiles, hormone levels, and the pathophysiology of brain injury^8^. Additionally, other factors, such as race and socioeconomic status, may also affect the sex disparity of IS^1^. A better understanding of sex disparities in IS may call for improved preventive and therapeutic strategies.

Disability-adjusted life years (DALYs) is a widely used metric to assess the disease burden integrating both mortality (years of life lost [YLLs]) and disability (years lived with disability [YLDs])^9^. The updated global burden of disease (GBD) 2019 study reported the DALYs number due to IS was increasing. However, discussion regarding the temporal and spatial trends in the sex distribution of IS DALYs is sparse. Therefore, we aimed to provide updated data on the sex difference in IS DALYs using GBD 2019 data.

## Methods

### Data sources

Data in this study were extracted from the Global Health Data Exchange (GHDx) query tool (http://ghdx.healthdata.org/gbd-results-tool), which provides an updated assessment of burden from diseases, injuries, and risk factors across the counties and territories. Measures included DALYs number, age-standardized DALYs rate (ASDR), all-age YLDs rate, all-age YLLs rate due to IS in 204 countries and territories and 21 regions from January 1st, 1990 to December 31st, 2019. We also collected data by level 2 risk factors and sociodemographic index (SDI), which ranges from zero to one, and is a composite measure of education, fertility and income per capita for a specific location-year. Countries and territories were classified into five regions by SDI, which were high, high-middle, middle, low-middle, low SDI regions. According to the sex, the study population was divided into both, me and women groups.

The ethics committee of the First Affiliated Hospital of Xi’an Jiaotong University confirmed that ethics approval was not required for this study because it was based on a publicly available GBD database and did not include identified personal information. The study followed the Creative Commons Attribution-NonCommercial 4.0 International License (https://creativecommons.org/licenses/by-nc/4.0/), which allows non-commercial users to organize, share, modify or build the data on IHME Websites. All procedures performed in this study involving human participants were in accordance with the ethical standards of the institutional research committee and with the Declaration of Helsinki.

### Statistical analysis

The detailed methods for the assessment of disease DALYs have been described previously^9,10^. Differences in DALYs number (men minus women) and ASDR (men to women’s ASDR ratio) were used to compare the relative levels of IS DALYS between sexes. We used percentage changes and estimated annual percentage change (EAPC) to assess the trends in DALYs number and ASDR. It is assumed that the natural logarithm of ASDR follows a regression line. Thus, Y = α + βX + ε, where Y denotes ln (ASDR), X means a calendar year, and ε refers to the error term. Herein, β determines the positive or negative trends of ASDR. Then, EAPC = 100*(exp(β)− 1). Its 95% confidence intervals are also obtained from the linear model. When the EAPC value and its upper boundary of the confidence interval are positive, the trend in ASDR is increasing. Instead, when the EAPC value and its lower boundary of the confidence interval are negative, the trend in ASDR is decreasing. Pearson correlation and linear regression analyses were conducted to identify the correlations between ASDR ratio and SDI. R software version 3.5.2 (https://cran.r-project.org/doc/FAQ/R-FAQ.html#Citing-R) and SPSS software version 21.0 (https://www.ibm.com/products/spss-statistics) were used for charting and analysis. A two-tailed *P* value less than 0.05 was considered statistically significant.

## Results

### Sex differences in global DALYs due to ischemic stroke

**Figure 1:**
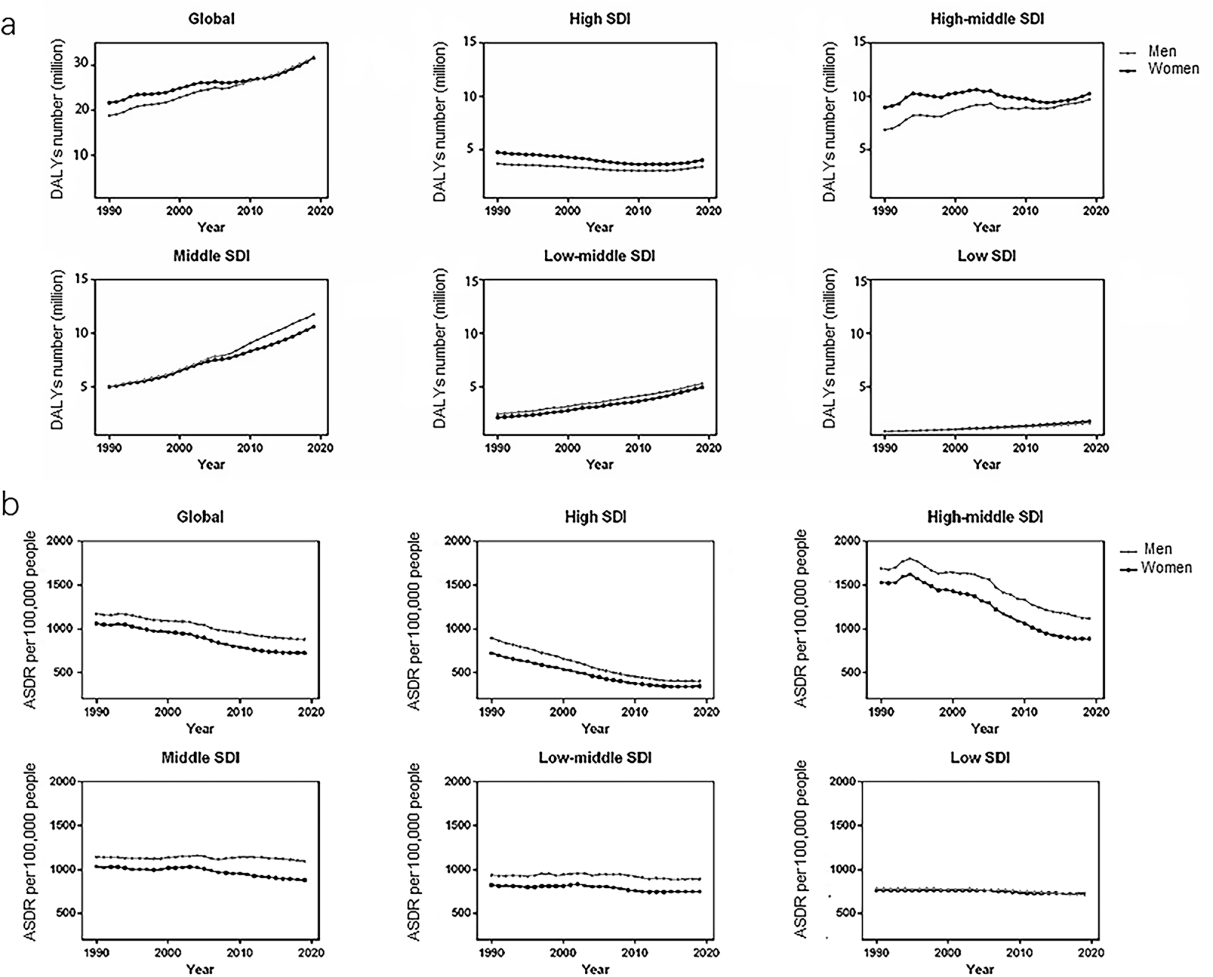
The sex-specific DALYs number (**a**) and ASDR (**b**) due to ischemic stroke in global and five SDI regions from 1990 to 2019. Abbreviations: DALYs, disability-adjusted life years; ASDR, age-standardized DALYs rate; SDI, socio-demographic index.

**Figure 2:**
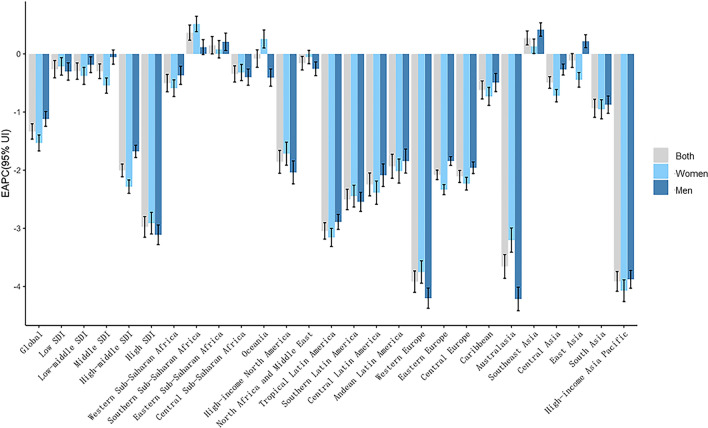
The sex-specific EAPC of ASDR due to ischemic stroke from 1990 to 2019, by sex and regions. Abbreviations: EAPC, estimated annual percentage change; ASDR, age-standardized DALYs rate; SDI, socio-demographic index.

**Figure 3:**
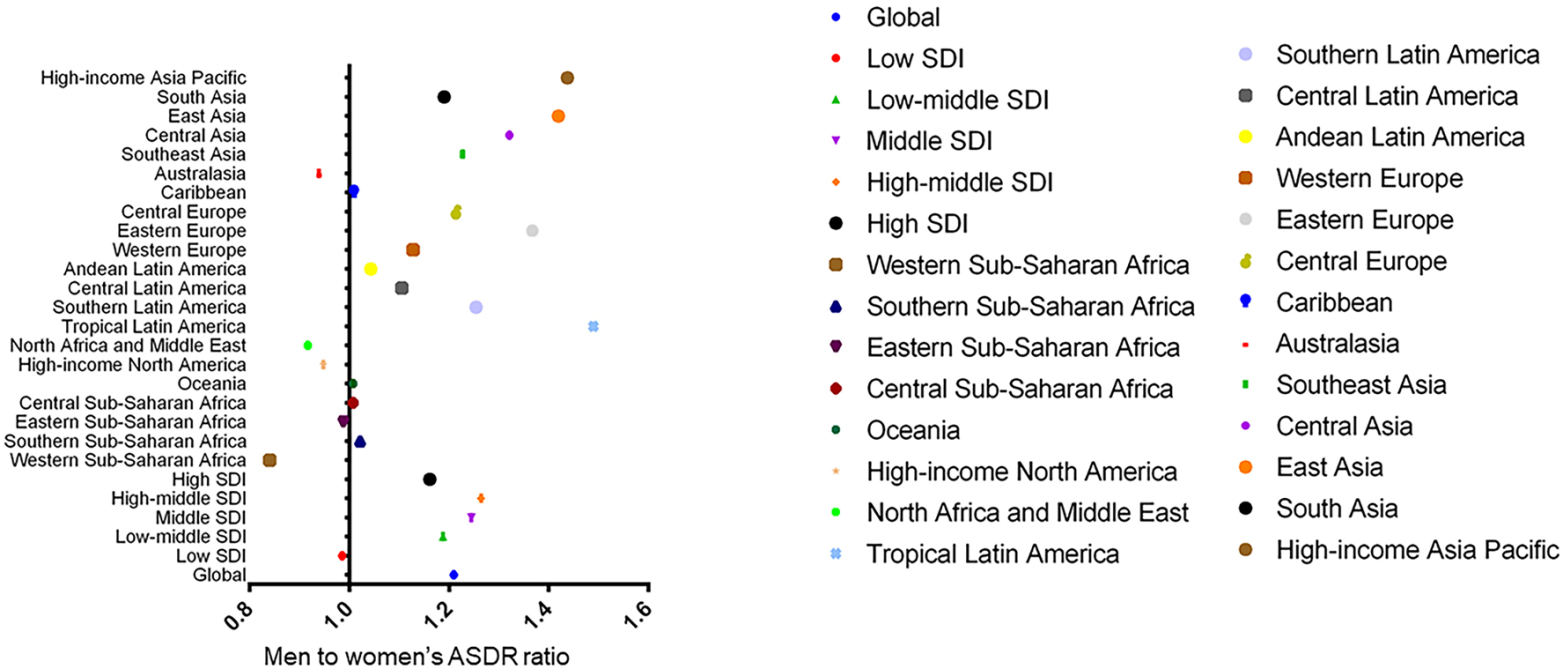
The men to women’s ASDR ratio in global, five SDI regions and 21 geographic regions. Abbreviations: ASDR, age-standardized DALYs rate; SDI, socio-demographic index.

During the past three decades, the global DALYs number of IS for men and women has increased by 72.44% and 49.78% respectively, but the absolute difference between sex (men minus women) has decreased from 2.83 million in 1990 to 0.14 million in 2019. (Table 1) (Fig. 1a). After adjustment for age structure and population size, the global ASDR of IS for men and women slightly decreased, with EAPC values of − 1.12 and − 1.53 respectively. (Figs. 1b and 2). The sex difference in ASDR of IS has persisted, and the men to women’s ASDR ratios slightly increased from 1.10 in 1990 to 1.21 in 2019 (Figs. 1b and 3).

**Table 1:** The sex-specific DALYs number and age-standardized DALYs rate due to ischemic stroke in 2019, and its temporal trends from 1990 to 2019.

Types	Both				Women				Men			
	DALYs number (million)	ASDR (per 100,000)	Change in absolute number (percent)	EAPC	DALYs number (million)	ASDR (per 100,000)	Change in absolute number (percent)	EAPC	DALYs number (million)	ASDR (per 100,000)	Change in absolute number (percent)	EAPC
Global	63.48(57.83–68.99)	798.81(727.51–866.89)	60.31(47.75–71.88)	− 1.33(− 1.47–− 1.2)	31.67(28.28–34.77)	726.33(648.67–798.32)	49.78(37.76–63.86)	− 1.53(− 1.67–− 1.39)	31.81(28.6–34.75)	878.51(793.52–956.67)	72.44(54.1–87.33)	− 1.12(− 1.25–− 0.99)
Low SDI	3.42(2.92–4.11)	720.34(610.62–867.08)	116.46(87.6–146.72)	− 0.26(− 0.41–− 0.11)	1.79(1.55–2.11)	724.97(624.94–848.09)	130.28(93.29–170.41)	− 0.21(− 0.37–− 0.06)	1.63(1.32–2.15)	714.67(575.68–941.18)	103.2(68.82–146.89)	− 0.3(− 0.45–− 0.15)
Low-middle SDI	10.22(9.16–11.37)	816.32(733.23–904.22)	167.59(135.52–200.85)	− 0.3(− 0.44–− 0.15)	4.95(4.36–5.55)	749.55(662.98–837.97)	179.71(143.78–230.83)	− 0.38(− 0.52–− 0.23)	5.28(4.51–6.13)	890.48(764.59–1027.23)	157.05(118.9–194.66)	− 0.19(− 0.32–− 0.05)
Middle SDI	22.39(20.04–24.77)	982.89(881.86–1084.73)	120.09(86.89–143.13)	− 0.3(− 0.43–− 0.17)	10.62(9.27–12.05)	880.44(767.96–995.53)	110.35(83.54–138.72)	− 0.55(− 0.68–− 0.41)	11.77(10.29–13.29)	1096.01(964.62–1224.57)	129.92(85.85–159.17)	− 0.05(− 0.18–0.07)
High-middle SDI	19.96(18.13–21.61)	993.5(901.35–1076.67)	20.41(12.46–27.27)	− 2(− 2.11–− 1.89)	10.25(9.11–11.28)	885.51(787.06–976.5)	7.67(0.8–15.37)	− 2.28(− 2.4–− 2.17)	9.71(8.72–10.73)	1119.44(1006.73–1234.25)	36.61(23.55–45.2)	− 1.68(− 1.78–− 1.57)
High SDI	7.46(6.57–8.25)	371.79(327.55–413.68)	− 26.05(− 29.6–− 22.86)	− 2.98(− 3.15–− 2.8)	4.04(3.46–4.51)	344.94(296.45–389.25)	− 29.94(− 33.98–− 26.22)	− 2.91(− 3.1–− 2.72)	3.41(3.08–3.74)	400.67(360.35–440.53)	− 21.36(− 24.94–− 17.95)	− 3.11(− 3.28–− 2.94)
Western Sub-Saharan Africa	1.23(1.05–1.44)	700.79(609.53–799.59)	57.06(30.44–85.45)	− 0.5(− 0.65–− 0.35)	0.69(0.59–0.82)	758.85(648.3–885.37)	54(25.73–84.27)	− 0.59(− 0.73–− 0.45)	0.53(0.44–0.67)	637.7(532.78–774.38)	60.42(28.72–99.17)	− 0.37(− 0.53–− 0.21)
Southern Sub-Saharan Africa	0.44(0.4–0.47)	883.87(803.05–956.67)	42.99(29.96–59.88)	0.37(0.24–0.5)	0.26(0.23–0.29)	870.27(778.27–961.09)	43.31(28.21–62.13)	0.51(0.38–0.65)	0.18(0.16–0.19)	889.26(812.02–965.88)	39.93(23–60.01)	0.12(− 0.01–0.25)
Eastern Sub-Saharan Africa	1.12(0.94–1.34)	773.6(651.24–904.39)	160.39(111.22–212.35)	0.15(0–0.3)	0.61(0.52–0.71)	775.93(656.37–900.1)	168.21(120.03–214.22)	0.08(− 0.07–0.23)	0.51(0.42–0.65)	766.69(617.02–974.62)	152.2(87.22–234.29)	0.21(0.06–0.36)
Central Sub-Saharan Africa	0.36(0.29–0.46)	831.15(658.32–1040.01)	109.02(78.4–145.3)	− 0.34(− 0.48–− 0.2)	0.21(0.16–0.26)	827.08(637.91–1046.61)	129.38(90.55–175.3)	− 0.32(− 0.46–− 0.18)	0.16(0.12–0.22)	833.48(653.17–1127.76)	87.36(52.42–128.74)	− 0.4(− 0.54–− 0.26)
Oceania	0.05(0.04–0.06)	741.87(610.33–931.42)	28.25(14.25–45.27)	− 0.08(− 0.23–0.07)	0.02(0.02–0.03)	738.28(613.8–897.31)	35.62(17.99–57.13)	0.26(0.1–0.41)	0.02(0.02–0.04)	743.93(577.38–1068.1)	21.79(5.22–41.51)	− 0.41(− 0.55–− 0.26)
High-income North America	2.26(1.96–2.54)	351.61(303.26–399.7)	− 18.14(− 21.52–− 14.31)	− 1.86(− 2.05–− 1.66)	1.32(1.14–1.49)	358.35(306.12–408.44)	− 21.97(− 25.51–− 18.23)	− 1.72(− 1.91–− 1.52)	0.94(0.82–1.06)	339.78(294.32–384.53)	− 13.36(− 18.28–− 8.3)	− 2.04(− 2.24–− 1.84)
North Africa and Middle East	4.75(4.23–5.27)	1183.57(1060.85–1307.04)	123.51(95.9–151.09)	− 0.16(− 0.28–− 0.04)	2.43(2.15–2.7)	1235.27(1100.81–1367.18)	128.23(96.78–159.25)	− 0.05(− 0.17–0.06)	2.33(2.04–2.65)	1132.06(996–1278.91)	118.85(86.78–156.07)	− 0.26(− 0.38–− 0.14)
Tropical Latin America	1.3(1.19–1.39)	561.21(511.41–599.69)	9.41(0.69–18.55)	− 3.04(− 3.19–− 2.9)	0.6(0.54–0.66)	460.68(410.53–504.06)	9.87(− 1.7–21.6)	− 3.16(− 3.31–− 3)	0.7(0.64–0.74)	686.39(629.64–733.5)	8.91(0.58–18.95)	− 2.89(− 3.02–− 2.76)
Southern Latin America	0.34(0.31–0.38)	404.42(366.65–440.48)	− 18.05(− 27.54–− 8.27)	− 2.5(− 2.68–− 2.33)	0.18(0.16–0.2)	363.28(321.73–401.45)	− 18.46(− 29.16–− 6.24)	− 2.45(− 2.63–− 2.26)	0.16(0.15–0.18)	455.71(413.54–496.01)	− 18.42(− 29.6–− 5.88)	− 2.54(− 2.71–− 2.38)
Central Latin America	0.77(0.68–0.87)	340.17(297.82–385.12)	34.54(22.75–48.29)	− 2.24(− 2.44–− 2.05)	0.4(0.35–0.46)	324.33(280.88–372.26)	29.92(16.93–45.53)	− 2.39(− 2.59–− 2.19)	0.37(0.32–0.42)	358.46(309.31–408.31)	38.54(23.68–52.61)	− 2.09(− 2.28–− 1.89)
Andean Latin America	0.17(0.15–0.2)	316.29(268.4–375.29)	76.7(48.71–112.91)	− 1.93(− 2.14–− 1.73)	0.09(0.07–0.11)	309.84(259.42–365.72)	79.84(48.62–117.08)	− 2.02(− 2.22–− 1.81)	0.08(0.07–0.1)	323.25(267.79–392.02)	73.18(45.17–132.94)	− 1.84(− 2.05–− 1.64)
Western Europe	3.24(2.86–3.51)	309.64(275.76–336.74)	− 38.57(− 42.14–− 35.62)	− 3.92(− 4.1–− 3.73)	1.84(1.59–2.03)	291.36(254.41–322.87)	− 41.03(− 45.07–− 37.65)	− 3.75(− 3.94–− 3.56)	1.39(1.27–1.49)	328.64(300.28–353.24)	− 35.82(− 39.08–− 32.61)	− 4.2(− 4.38–− 4.03)
Eastern Europe	5.82(5.23–6.41)	1667.98(1502.07–1837.63)	− 11.55(− 16.13–− 7.6)	− 2.08(− 2.16–− 2)	3.37(2.95–3.81)	1447.36(1269.23–1640.56)	− 18.79(− 24.36–− 14.76)	− 2.33(− 2.42–− 2.25)	2.45(2.13–2.79)	1978.65(1724.07–2242.97)	2.01(− 4.51–8.13)	− 1.84(− 1.92–− 1.77)
Central Europe	2.42(2.12–2.71)	1087.72(953.98–1213.31)	6.18(− 1.42–11.78)	− 2.11(− 2.21–− 2)	1.35(1.18–1.5)	990.08(864.72–1104.55)	1.71(− 5.66–8.02)	− 2.23(− 2.34–− 2.12)	1.07(0.94–1.21)	1201.52(1056.57–1347.76)	10.01(1.36–16.72)	− 1.96(− 2.06–− 1.86)
Caribbean	0.34(0.3–0.39)	662.02(575.72–760.2)	46.09(34.23–60.47)	− 0.62(− 0.78–− 0.47)	0.18(0.16–0.21)	658.04(567.4–759.21)	47.11(33.82–63.88)	− 0.73(− 0.89–− 0.58)	0.16(0.13–0.18)	664.2(569.24–778.16)	44.91(30.5–59.97)	− 0.49(− 0.65–− 0.34)
Australasia	0.14(0.12–0.16)	260.33(226.32–290.68)	− 29.3(− 34.29–− 24.68)	− 3.66(− 3.86–− 3.45)	0.08(0.07–0.09)	266.54(226.93–303.34)	− 30.02(− 35.27–− 24.71)	− 3.2(− 3.41–− 2.99)	0.06(0.05–0.06)	250.31(223.15–276.35)	− 29.87(− 35.62–− 24.05)	− 4.21(− 4.42–− 4.01)
Southeast Asia	6.33(5.44–7.13)	1175.56(1018.24–1313)	156.8(127.82–188.99)	0.27(0.15–0.39)	3.13(2.7–3.58)	1061.94(918.95–1211.34)	155.7(123.81–197.81)	0.13(0.01–0.26)	3.2(2.6–3.75)	1303.35(1074.27–1509.47)	158.11(121.08–193.05)	0.42(0.3–0.53)
Central Asia	0.88(0.81–0.97)	1386.79(1269.8–1515.23)	34.92(16.84–52.53)	− 0.49(− 0.59–− 0.39)	3.63(3.02–4.28)	1220.04(1102.14–1352.47)	20.5(1.98–36.8)	− 0.72(− 0.83–− 0.61)	4.11(3.23–5.18)	1611.63(1473.26–1759.08)	53.96(34.08–77.02)	− 0.27(− 0.36–− 0.17)
East Asia	21.98(19.27–24.99)	1135.03(997.93–1284.34)	153.67(109.08–181.15)	− 0.11(− 0.23–0.01)	9.92(8.26–11.67)	958.02(801.22–1119.73)	143.79(111.03–177.29)	− 0.45(− 0.57–− 0.32)	12.06(10.06–14.29)	1359.64(1141.68–1589.66)	163.31(100.03–199.96)	0.22(0.11–0.33)
South Asia	7.75(6.61–9.08)	605.29(521.1–706.71)	156.58(116.35–198.81)	− 0.94(− 1.09–− 0.78)	0.44(0.4–0.49)	554.09(461.09–648.14)	182.96(132.63–260.46)	− 0.95(− 1.12–− 0.79)	0.44(0.4–0.49)	659.45(522.14–824.57)	136.65(95.81–182.35)	− 0.87(− 1.02–− 0.72)
High-income Asia Pacific	1.8(1.52–2.03)	352.82(303.27–401.33)	− 13.36(− 20.07–− 6.62)	− 3.91(− 4.08–− 3.74)	0.92(0.75–1.06)	294.43(243.82–343.21)	− 15.64(− 24.12–− 7.98)	− 4.07(− 4.26–− 3.88)	0.88(0.77–0.97)	423.2(372.92–471.5)	− 11.31(− 17.32–− 1.22)	− 3.88(− 4.03–− 3.72)

Abbreviations: DALYs, disability-adjusted life years; ASDR, age-standardized DALYs rate; SDI, socio-demographic index; EAPC, estimated annual percentage change.

**Figure 4:**
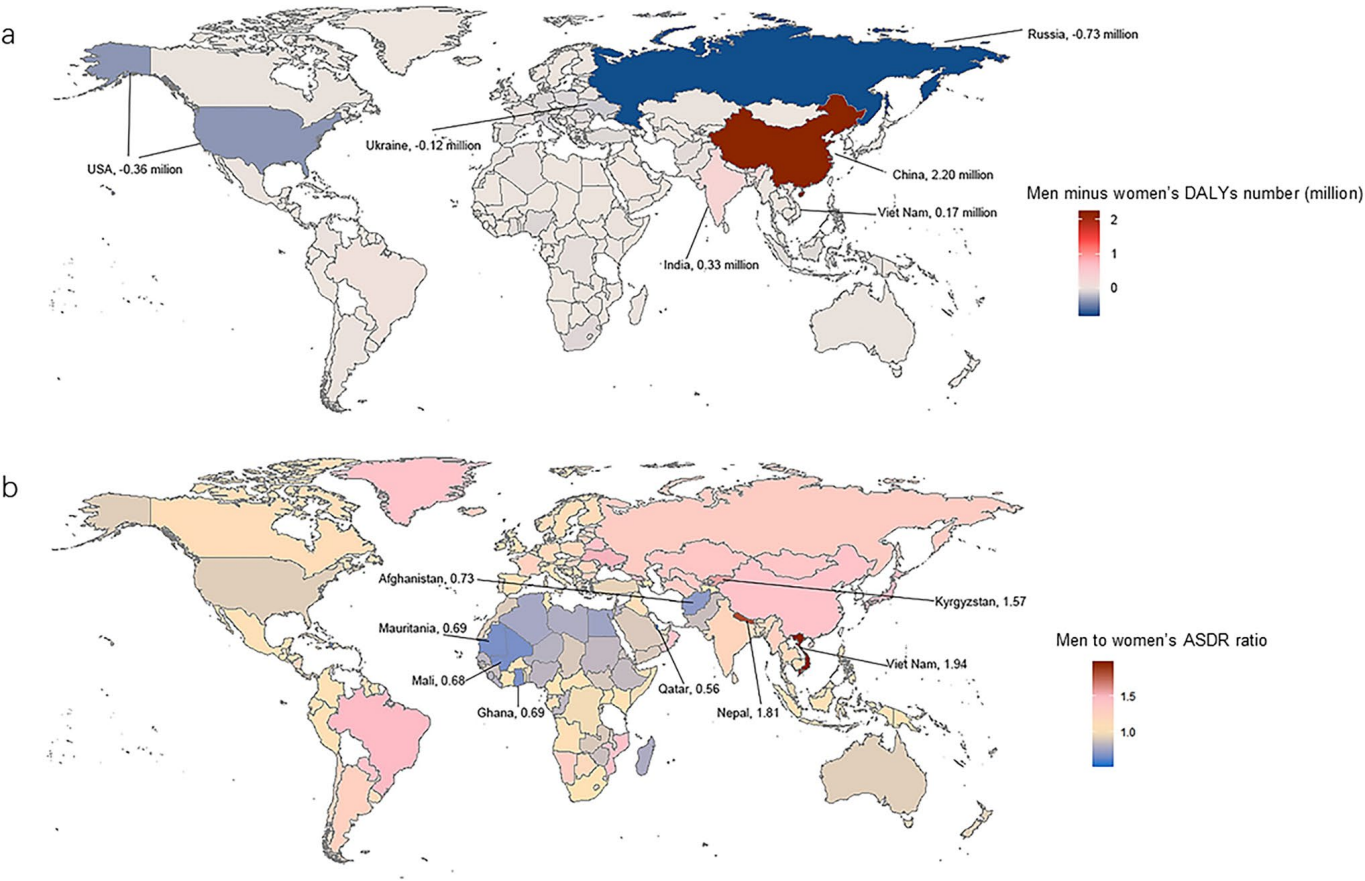
Global sex difference in DALYs number (men minus women) (**a**) and ASDR ratio (men to women) (**b**) due to ischemic stroke in 2019. Abbreviations: DALYs, disability-adjusted life years; ASDR, age-standardized DALYs rate.

Regional disparities have been observed regarding sex differences in IS DALYs. In 2019, women had a higher DALYs number than men in most regions except East Asia, South Asia, Tropical Latin America, Southeast Asia, and Oceania. The largest gap in the DALYs number between sexes moved from Eastern Europe with a difference (men minus women) of − 1.90 million in 1990 to East Asia with a difference of 2.15 million in 2019 (Table 1). After adjustment for age structure and population size, men had a higher ASDR in most regions except for Western Sub-Saharan Africa, North Africa and the Middle East, High-income North America, and Australasia. The greatest men to women’s ASDR ratio was found in Tropical Latin America (ASDR ratio = 1.49), followed by High-income Asia Pacific (ASDR ratio = 1.44), and East Asia (ASDR ratio = 1.42) (Fig. 3). Furthermore, a similar temporal trend of ASDR between sexes was observed in most regions except for Oceania (EAPC − 0.41, 95% CI − 0.55 to − 0.26 in men vs. 0.26, 95% CI 0.10 to 0.41 in women) and East Asia (EAPC 0.22, 95% CI 0.11 to 0.33 in men vs. − 0.45, 95% CI − 0.57 to − 0.32 in women) (Fig. 2). At the national level, the largest sex difference in DALYs number was found in China, with an estimated DALYs number of 2.2 million more in men than women (Fig. 4a, Supplementary Table S1). However, after adjustment, Vietnam (ASDR ratio = 1.94) and Qatar (ASDR ratio = 0.56) had the most significant sex difference in the ASDR of IS. (Fig. 4b, Supplementary Table S1).

### Sex difference in DALYs due to ischemic stroke by SDI

We further explored the association of SDI and sex differences in IS DALYs. Significant sex differences in DALYs number were found in the middle SDI region, with a higher number in men, as well as high and high-middle SDI regions, but with a higher number in women. During the past three decades, these gaps have widened in the middle SDI region but narrowed in the high and high-middle SDI regions. After adjustment, a higher ASDR of IS in men was found in almost all SDI regions (Figs. 1 and 3). Only the high SDI region showed a reduced difference by sex, with men to women’s ASDR ratios from 1.24 in 1990 to 1.16 in 2019 (Fig. 1). Furthermore, women had a significant reduction in ASDR compared with men in high-middle and middle SDI regions, and their 95% confidence intervals didn’t overlap (Fig. 1). Pearson correlation analyses suggested that the men to women’s ASDR ratio was weakly and positively correlated with SDI (r = 0.263; *P* < 0.001) (Supplementary Figure S1).

### Sex difference in DALYs due to ischemic stroke by age

**Figure 5:**
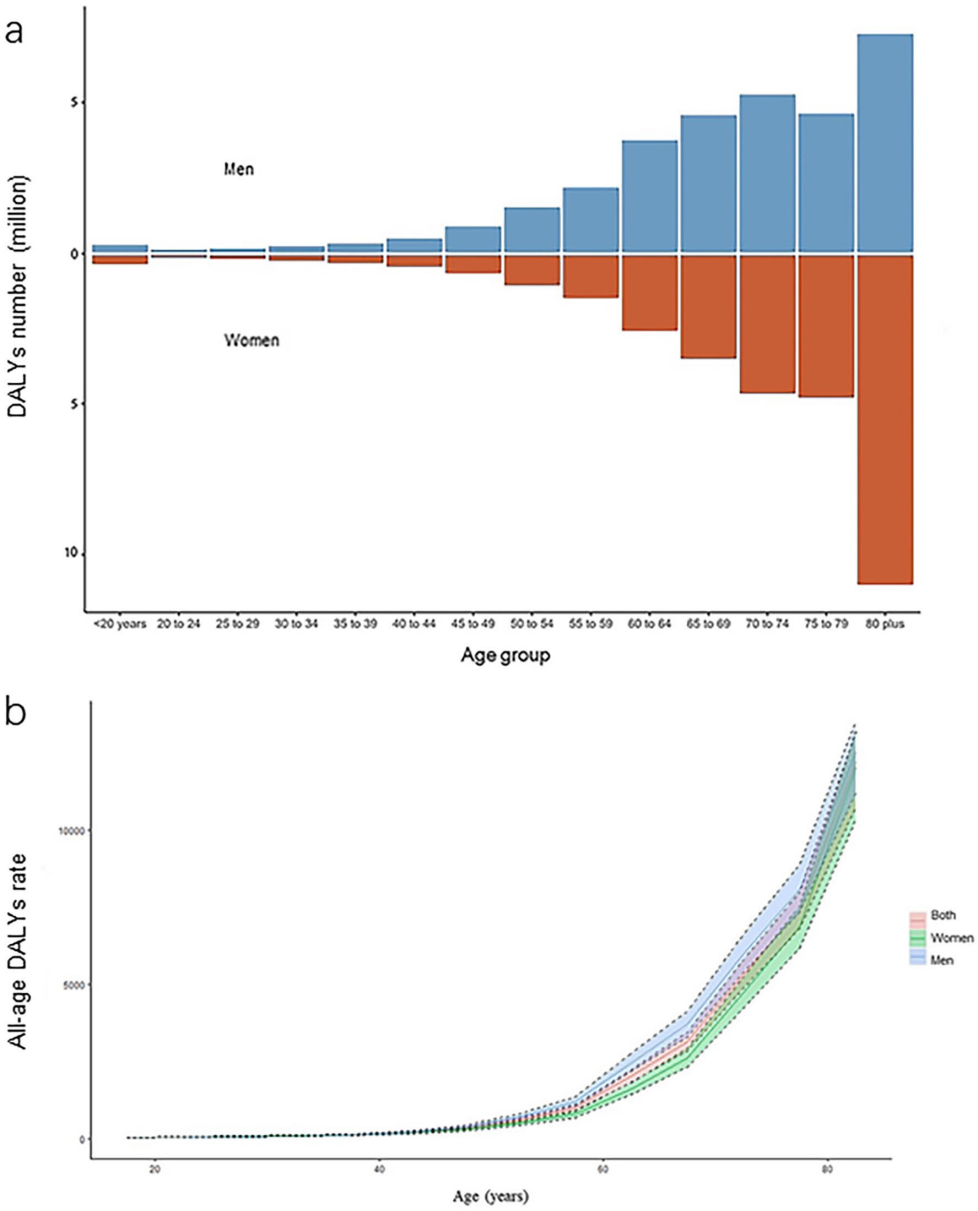
The sex-specific DALYs number (**a**) and all-age DALYs rate (**b**) due to ischemic stroke by age in 2019. Abbreviations: DALYs, disability-adjusted life years.

Age plays a critical role in the outcome of IS. Generally, DALYs number and all-age DALYs rate increased with age, but the sex differences in the above metrics varied across different age groups (Fig. 5a,b). Before 40 years old, sex differences in IS DALYs can be almost negligible. At middle age, men had a higher DALYs number as well as DALYs rate of IS than that of women. In regard to old age, these differences gradually vanished in the DALYs rate and even reversed in the DALYs number in individuals older than 75 years old. Given that DALYs is the composite of YLDs and YLLs, we investigate the sex differences in these two components by age. The results showed that men tended to have a higher all-age DALYs rate and YLLs rate, as women of the same age tended to have a higher all-age YLDs rate. Moreover, sex differences in the DALYs rate by age seemed to be largely influenced by the YLLs rate (Supplementary Figure S2).

### Sex differences in the attributable DALYs number due to ischemic stroke for twelve risk factors

**Figure 6:**
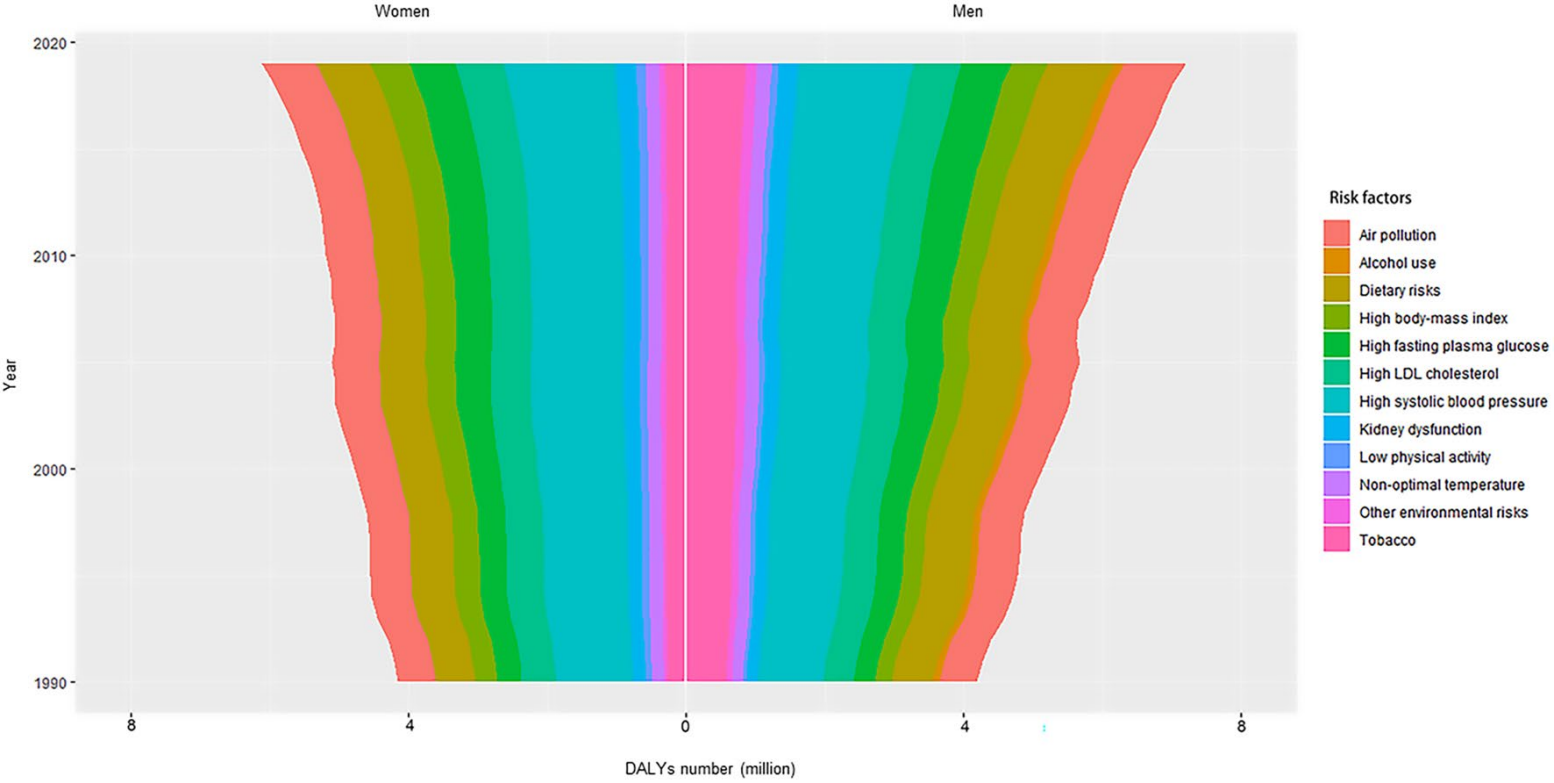
The sex-specific DALYs number due to ischemic stroke attributed to the twelve level 2 risk factors from 1990 to 2019. Abbreviations: DALYs, disability-adjusted life years.

Figure 6 indicates the sex differences in the global attributable DALYs number of IS for twelve risk factors. In 2019, risk factors including low physical activity, high body-mass index, high LDL cholesterol, and kidney dysfunction conferred more DALYs number for women, while the remains conferred more DALYs number for men. Notably, the most significant sex differences were observed in some unhealthy habits in 2019, such as tobacco (men minus women: 5.49 million), dietary risks (men minus women: 1.79 million) and alcohol use (men minus women: 1.71 million). The attributable DALYs number increased more in men than women from 1990 to 2019, and the most significant increase was found in high fasting plasma glucose for both sexes.

## Discussion

Our study provides the latest overview of sex differences in global DALYs due to IS. Globally, women accounted more DALYs number before 2012, and then remained at a similar level as men. After eliminating the effects of population size and age structure, sex differences in IS DALYs persisted from 1990 to 2019, always with a higher ASDR in men than women. In addition, sex differences in DALYs due to IS varied by year, location, SDI and age, as well as the related risk factors.

The global burden of IS DALYs for both sexes increased, which was largely driven by population growth and aging^10^. Meanwhile, the risk of IS mortality and disability increased with age. Considering that women had a longer life expectancy than men, more women lived with an increased risk of poor prognosis of IS, which might be one reason for the more DALYs number in women previously. The similar levels of DALYs number across sexes in recent years may result from the lower ASDR and a greater decreasing trend in women, also implying that women may benefit more from the current preventive measures and management of IS than men. Other factors including sex hormones, genetic factors, comorbidities or risk factors, ethnicity/race, and socioeconomic status, have been mentioned.

Geographical variations in sex differences in IS DALYs were noted in the present study and the reason needs to be considered from various perspectives. To some extent, socioeconomic status is a comprehensive reflection of the national healthcare system and vascular risk factors. In areas with lower socioeconomic status, individuals failed to receive timely and effective treatment post IS, leading to a high risk of stroke mortality^11^. Nevertheless, in our results, the greatest burden of IS DALYs was not found in low or low-middle SDI region, but in regions with middle and high-middle SDI, which also possess significant sex differences in IS ASDR, with a higher ASDR in men. This phenomenon is likely due to the large population size and high prevalence of vascular risk factors in these less wealthy regions^12^. Despite the medical intervention has improved because of economic growth, the primary prevention of IS is insufficient in these areas. This would be more significant for some behavioral risk factors such as tobacco and alcohol use, particularly among men. Studies have reported that IS deaths due to tobacco use are highest in China, Russia, and India^13^. Heavy drinking in Bulgaria is related to the high death number of ischemic heart disease^14^. High proportions of current smokers and heavy drinking in men were reported in Vietnam^15^, which has the greatest men to women’s ASDR ratio due to IS in our study. These sex-specific risk factors may make for the differences in IS DALYs between sexes. In regions below middle SDI, the low IS DALYs may be attributed to fewer vascular risk factors. In contrast, in high SDI region, it attributes to the advanced management of IS and effective prevention of vascular risk factors^16^. However, socioeconomic status cannot fully explain the geographical variations in sex differences in IS DALYs. For example, a significantly higher ASDR in men was noted in the high-income Asia Pacific region (ASDR ratio: 1.44) but not in high-income North America (ASDR ratio: 0.95). A previous study by Ayala et al. examined the sex difference in IS mortality by ethnicity/race, including Hispanics, non-Hispanics (American Indians and Alaska Natives, Asian Americans and Pacific Islanders, whites, and blacks), illustrating that men had a significantly higher risk of IS death than women in Asian Americans and Pacific Islanders (women to men’s rate ratios [RR] 0.74, 95% CI 0.71–0.78), whereas the risk was higher for women in non-Hispanic whites^1^. Other factors including diet habits, genetic and environmental factors have been mentioned^1,17,18^.

Age is another critical factor that affects sex differences in IS DALYs. Older patients with IS often have poor outcomes due to more comorbidities (such as hypertension, diabetes, or atrial fibrillation), more severe symptoms, more medical complications (such as infections), and less effective treatment compared with younger patients^19,20^. Our results showed that approximately 43.6% (27.69 million) of DALYs number due to IS occurred in individuals older than 75 years old. Among them, the greater DALYs number in women (15.80 million) may result from a longer life expectancy than men (11.90 million). Additionally, sex differences in DALYs number and DALYs rate by age were roughly similar, with a significant men’s predominance at middle age. This finding is consistent with a previous study showing that men aged 45–74 years old have higher stroke mortality than women’s counterparts in the USA^21^. Experimental studies also demonstrated that young female animals suffer smaller brain infarcts and fewer neurological deficits after stroke than age-matched male animals, but this protection disappears and is even worse in aged female animals. Age-related changes in sex hormones, especially estrogen, which provide a neuroprotective effect for women in different ways, play an essential role in this process^8^. In addition, changes in the risk factor profiles with age may also be associated with sex differences in IS DALYs. Hypertension is more common in young men and women over 65 years old^22^. The prevalence of atrial fibrillation, which is the leading cause of cardioembolic IS, increases dramatically with age. Women confer a higher risk of stroke and poor outcome than men in patients with atrial fibrillation^23,24^.

Except for demography and healthcare systems, it seemed that most variations in sex differences in IS DALYs could be explained by the disproportion of cardiovascular risk factors between sexes. In our findings, women had more IS DALYs number attributed to low physical activity, high body-mass index, high LDL cholesterol, and kidney dysfunction, but the largest sex differences were found in tobacco, dietary risk, and alcohol use, with more DALYs number in men. The literature points out that the social roles of men and women can affect not only the prevention of cardiovascular risk factors, but also the outcome of diseases^25,26^. Women tend to have more depression and less freedom to go outside due to family care responsibility, less financial decision-making power, or religious reasons, which may be associated with unhealthy diet habits and low physical activity, indirectly leading to high rates of obesity, hyperlipidemia, or metabolism syndrome. For example, unhealthy diet and physical inactivity, especially for women, are the two crucial threats in some Arab countries, which may be the cause of a higher IS ASDR for women in North Africa and Middle East^27,28^. In contrast, men tend to show their masculinity by some behaviors such as smoking or drinking, which are correlated with the sex difference in smoke mortality^29^. Additionally, they tend to have fewer healthcare-seeking behaviors and worse compliance than women. Hypertension is the leading cause of IS DALYs. Evidence has shown that women are more likely to receive antihypertension medications and benefit more from treatment than men, which might be the other reason for the higher ASDR in men^30^. All of these factors might be the causes of sex differences in IS DALYs, but the degree of each impact needs to be evaluated in the future.

## Limitations

Our study had several limitations as GBD study^9,10^. First, the uncertainty interval of DALYs might be underestimated, which may result from assumption that uncertainty is independent between YLL and YLD. Second, bias might exist, although the GBD study provides a high-quality estimate of global IS. In some low-income countries, missing data may lead to the underestimated burden of IS and sex differences in IS DALYs need to be reconsidered. The conclusions regarding the sex difference in IS DALYs may not apply to all districts within a country because the GBD estimations are based on national datasets. Third, some important risk factors for IS, such as atrial fibrillation and congenital heart disease, are not included in GBD data, thus we cannot analyze the sex distribution of IS DALYs attributed to these factors.

## Conclusion

Although the global DALYs number of IS was equivalent between sexes in 2019, men still had a higher ASDR than women. Meanwhile, sex differences in IS DALYs varied by year, location and age, mostly can be attributed to the disproportionality of cardiovascular risk factors between sexes. Considering population growth and aging, it is necessary to monitor sex differences in IS DALYs, and thus propose more individualized prevention and management measures for local administration to reduce the burden of IS.

## Supplementary Information

Supplementary Information.

Supplementary Figure S1.

Supplementary Figure S2.

Supplementary Table S1.

## Data Availability

The data used in this study is publicly available on the website (http://ghdx.healthdata.org/gbd-results-tooldata.org/gbd-results-tool). All data used in this study is publicly available.

## Abbreviations

DALYs: Disability-adjusted life years
IS: Ischemic stroke
ASDR: Age-standardized DALYs rate
GBD: Global burden of disease
EAPC: Estimated annual percentage change
SDI: Socio-demographic index
YLLs: Years of life lost
YLDs: Years lived with disability
