# Supplementary material for: Sex differences in global disability-adjusted life years due to ischemic stroke: findings from global burden of diseases study 2019
Source: Sci Rep. 2022 Apr 14;12:6235. doi: 10.1038/s41598-022-10198-9 (PMC9010406; doi:10.1038/s41598-022-10198-9)
Supplement: Supplementary file 1 — Supplementary Information. [file 41598_2022_10198_MOESM1_ESM.docx]

Title: Sex differences in global disability-adjusted life years due to ischemic stroke: findings from global burden of diseases study 2019

Author list

Miaomiao Cao1†, MD., Bolin Li1†, Jie Rong2, MD., Qian Li1, MD., Chaofeng Sun1*, MD., PhD.

1 Department of Cardiology, First Affiliated Hospital of Xi'an Jiaotong University, Institute of Cardiovascular Channelopathy, Key Laboratory of Molecular Cardiology, Xi'an, Shaanxi 710061, P.R. China

2 Department of Encephalopathy, Affiliated Hospital of Shaanxi University of Chinese Medicine, Xianyang, China

†These authors have contributed equally to this work

*Correspondence to the author:

Professor Chaofeng Sun, Department of Cardiology, First Affiliated Hospital of Xi'an Jiaotong University, Institute of Cardiovascular Channelopathy, Key Laboratory of Molecular Cardiology, Xi'an, Shaanxi 710061, P.R. China

E‑mail: cfsun123@126.com

Figure legends

Figure S1. The relationship between men to women’s ASDR ratio and SDI at the national level. The lines indicate linear fit; data points, countries with corresponding SDI and men to women’s ASDR ratio. Abbreviations: ASDR, age-standardized DALYs rate; SDI, socio-demographic index.

Figure S2. The sex-specific YLDs rate (a) and YLLs rate (b) due to ischemic stroke by age in 2019. Abbreviations: YLL, years of life lost; YLD, years lived with disability.
