# Supplementary figures and images for: Sex differences in global disability-adjusted life years due to ischemic stroke: findings from global burden of diseases study 2019
Source: Sci Rep. 2022 Apr 14;12:6235. doi: 10.1038/s41598-022-10198-9 (PMC9010406; doi:10.1038/s41598-022-10198-9)

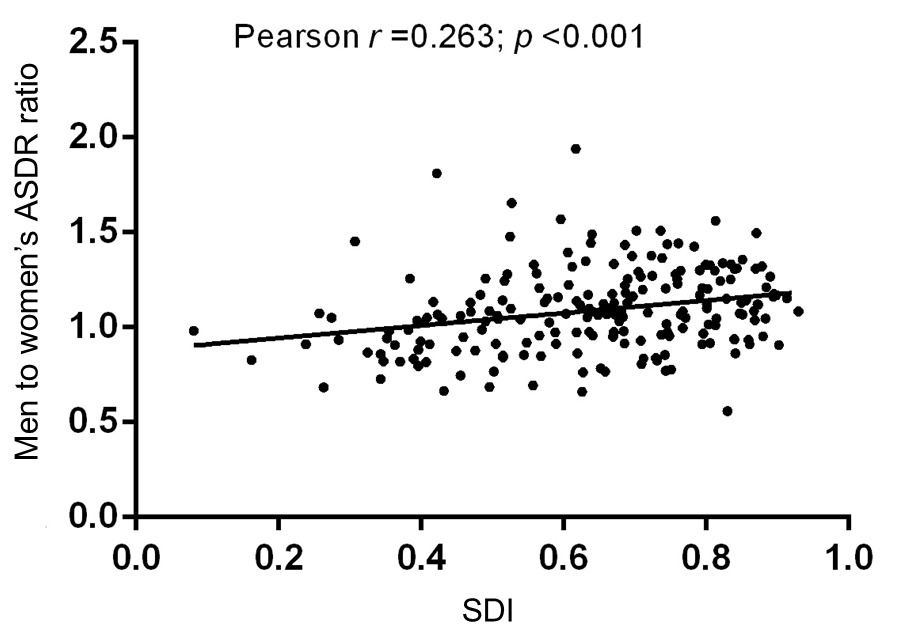

Supplement: Supplementary file 2 — Supplementary Figure S1. [file 41598_2022_10198_MOESM2_ESM.jpg]

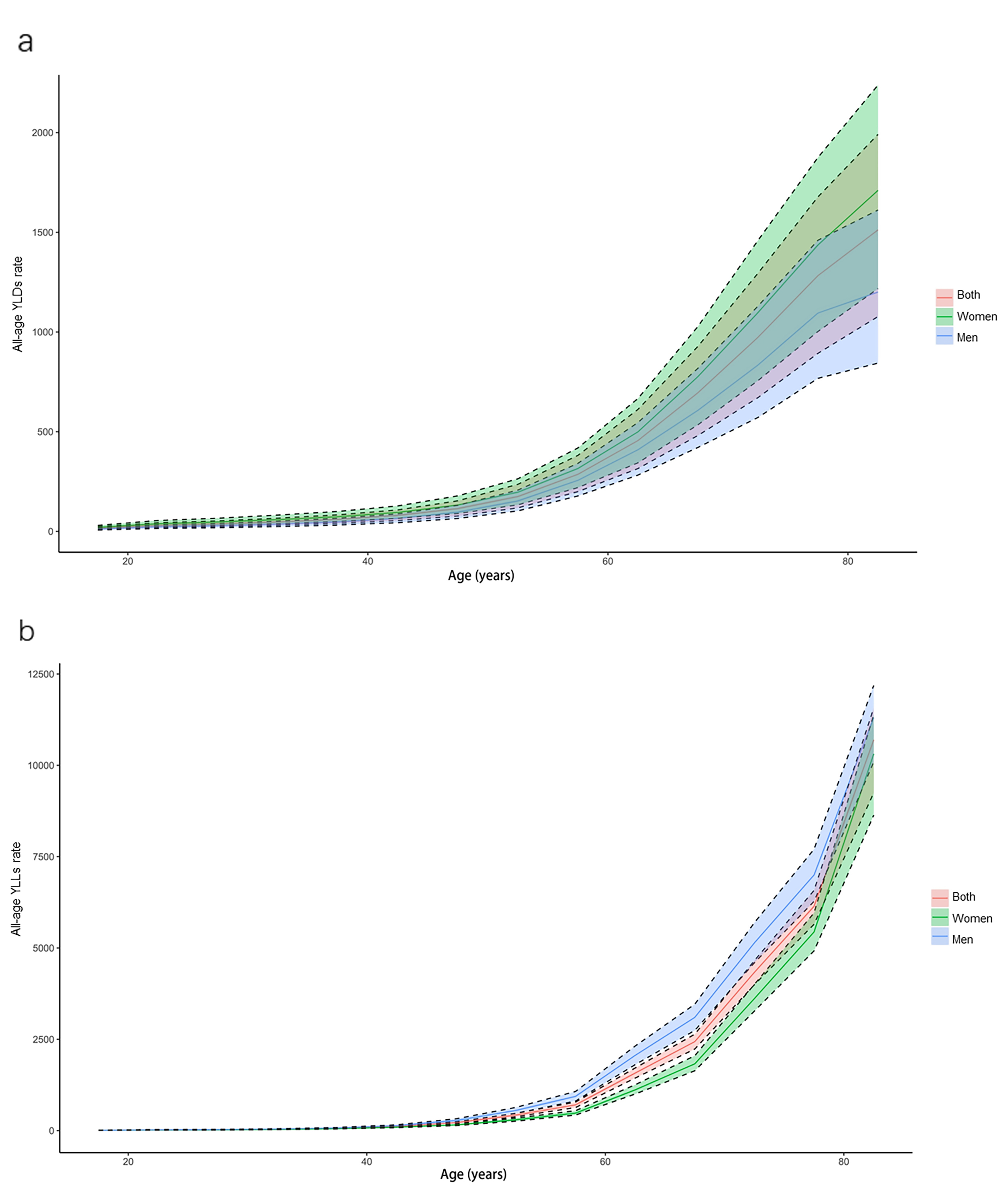

Supplement: Supplementary file 3 — Supplementary Figure S2. [file 41598_2022_10198_MOESM3_ESM.jpg]
