# Supplementary material for: Sex differences in global disability-adjusted life years due to ischemic stroke: findings from global burden of diseases study 2019
Source: Sci Rep. 2022 Apr 14;12:6235. doi: 10.1038/s41598-022-10198-9 (PMC9010406; doi:10.1038/s41598-022-10198-9)
Supplement: Supplementary file 4 — Supplementary Table S1. [file 41598_2022_10198_MOESM4_ESM.docx]

Table S1. The sex-specific DALYs number and age-standardized DALYs rate due to ischemic stroke in 2019, and its temporal trends from 1990 to 2019 at the countries and territories.

| Countries and territories | Both | | | | Women | | | | Men | | | |
| --- | --- | --- | --- | --- | --- | --- | --- | --- | --- | --- | --- | --- |
|  | DALYs number | ASDR (per 100 000 people) | Change in absolute number (percent) | EAPC | DALYs number | ASDR (per 100 000 people) | Change in absolute number (percent) | EAPC | DALYs number | ASDR (per 100 000 people) | Change in absolute number (percent) | EAPC |
| Afghanistan | 224570.42(170361.36–298131.18) | 1865.52(1381.78–2444.22) | 61.13(25.86–99.64) | 1.02(0.92–1.12) | 136236.99(100366.13–184304.45) | 2151.7(1535.71–2867.66) | 85.51(40.55–133.55) | 1.14(1.05–1.24) | 88333.43(64414.92–122448.7) | 1567.28(1148.72–2085.13) | 34.16(-0.51–80.14) | 0.82(0.71–0.92) |
| Albania | 23509.34(18260.17–29477.81) | 562.13(441.56–700.27) | 125.45(88.93–158.1) | -0.24(-0.41–-0.07) | 12072.15(9623.71–14791.47) | 541.35(434.08–659.96) | 118.15(86.71–153.71) | -0.21(-0.38–-0.03) | 11437.2(8270.22–15149.54) | 588.17(430.4–777.19) | 132.72(79.38–177.85) | -0.3(-0.47–-0.13) |
| Algeria | 372748.77(302682.81–448803.27) | 1275.12(1052.75–1530.17) | 117.95(82.9–156.04) | -0.85(-0.96–-0.74) | 198670.36(161395.67–237525.05) | 1457.09(1172.11–1732.51) | 110.6(73.87–152.4) | -0.56(-0.66–-0.46) | 174078.4(134080.53–219863.18) | 1143.21(889.84–1430.47) | 123.69(79.25–180.99) | -1.1(-1.21–-0.99) |
| American Samoa | 316.68(270.9–362.35) | 731.46(627.08–834.1) | 40.93(23.23–60.46) | -0.73(-0.88–-0.59) | 178.93(149.5–210.15) | 789.38(660.56–928.45) | 49.66(29.76–72.85) | -0.33(-0.48–-0.18) | 137.75(116.32–158.59) | 661.04(565.04–755.92) | 28.15(4.26–56.7) | -1.2(-1.35–-1.05) |
| Andorra | 352.12(284.98–429.34) | 241.61(196.79–295.55) | 2.22(-12.31–19.3) | -2.11(-2.34–-1.87) | 176.81(138.4–225.44) | 224.06(176.54–286.56) | 8.96(-11.72–33.04) | -1.96(-2.21–-1.71) | 175.31(143.23–218.63) | 260.43(212.26–323.96) | -4.07(-18.94–14.39) | -2.19(-2.41–-1.97) |
| Angola | 83386.3(70155.54–100432.77) | 916.59(775.32–1101.85) | 167.84(117.22–230.09) | -0.13(-0.26–0.01) | 44819.32(36819.38–55550.71) | 884.56(720.59–1091.51) | 187.05(121.62–270.5) | -0.08(-0.21–0.06) | 38566.98(31189.6–48881.41) | 956.89(795.92–1194.79) | 147.68(84.47–230.38) | -0.15(-0.28–-0.01) |
| Antigua | 519.93(454.28–588.68) | 573.39(500.52–648.75) | -21.26(-30.79–-11) | -1.44(-1.6–-1.28) | 316.78(274.33–360.42) | 639.26(556.94–723.2) | -19.53(-30.27–-7.57) | -0.95(-1.1–-0.79) | 203.15(172.07–235.9) | 493.08(420.65–573) | -25.41(-36.4–-11.96) | -2.07(-2.24–-1.91) |
| Argentina | 209972.71(187084.88–231959.14) | 379.29(337.67–418.64) | -22.93(-33.43–-9.62) | -2.57(-2.76–-2.39) | 111831.68(98286.24–125343.34) | 639.26(556.94–723.2) | -21.93(-33.97–-5.33) | -0.95(-1.1–-0.79) | 98141.03(86788.98–109782.54) | 430.28(381.33–480.01) | -24.66(-37.83–-8.91) | -2.66(-2.83–-2.49) |
| Armenia | 31329.31(27048.05–36175.96) | 774(669.57–891.51) | 15.3(0.42–34.61) | -2.6(-2.72–-2.48) | 17466.92(14979.98–20276.01) | 339.26(297.07–381.8) | 7.36(-7.83–25.98) | -2.46(-2.66–-2.27) | 13862.39(11778.78–16106.59) | 825.03(703.35–953.84) | 24.12(6.19–49.67) | -2.59(-2.71–-2.48) |
| Australia | 114238.13(98585.57–127019.11) | 252.75(218.67–282.92) | -31.12(-36.41–-26.19) | -3.75(-3.95–-3.54) | 66322.78(55671.05–74949.78) | 730.2(628.28–845.6) | -31.49(-37.26–-26.01) | -2.65(-2.77–-2.53) | 47915.35(42312.52–53213.18) | 243.09(214.15–270.02) | -32.16(-38.28–-25.72) | -4.33(-4.53–-4.12) |
| Austria | 53126.37(46623.7–58823.91) | 268.44(236.34–298.48) | -54.9(-58.39–-51.61) | -4.85(-5.04–-4.67) | 30184.22(25752.4–33856.94) | 258.81(219.25–294.33) | -57.26(-61.1–-53.7) | -3.28(-3.49–-3.06) | 22942.16(20430.17–25337.53) | 285.98(254.33–315.57) | -51.2(-55.85–-46.73) | -5.22(-5.4–-5.05) |
| Azerbaijan | 76935.29(63353.39–90322.05) | 1047.83(857.53–1221.01) | 122.19(83.41–162.56) | 1.77(1.63–1.9) | 41477(34264.94–49702.55) | 252.95(216.77–286.29) | 111.5(73.52–157.4) | -4.67(-4.86–-4.47) | 35458.29(25932.44–44897.11) | 1077.31(767.65–1354) | 135.25(79.02–194.27) | 1.6(1.47–1.74) |
| Bahamas | 1799.87(1519.38–2132.12) | 513.62(437.44–605.07) | 7.04(-5.46–21.3) | -1.17(-1.34–-1) | 1013.93(863.91–1194.08) | 1020.38(847.28–1218.66) | 5.03(-8.57–19.23) | 1.86(1.72–1.99) | 785.94(646.43–929.59) | 502.62(415.87–590.49) | 10.13(-6.61–30.32) | -1.34(-1.52–-1.17) |
| Bahrain | 4155.44(3481.99–5019.31) | 613.59(521.71–746.7) | 15.71(1.1–32.52) | -1.93(-2.07–-1.78) | 1897.02(1590.91–2263.75) | 519.41(441.25–610.68) | 9.3(-6.7–29.19) | -1.04(-1.21–-0.87) | 2258.43(1848.77–2811.32) | 543.05(449.64–724.86) | 24.41(5.41–47.27) | -1.76(-1.91–-1.61) |
| Bangladesh | 1056724.08(793190.71–1381291.29) | 898.24(676.58–1159.08) | 370.82(261.22–479.32) | 0.16(0.03–0.29) | 451493.42(345203.85–572870.87) | 697.94(589.49–827.02) | 376.41(267.23–515.91) | -2.01(-2.14–-1.88) | 605230.66(413855.95–873338.81) | 966.15(665.07–1367.37) | 368.63(234.67–519.32) | 0.18(0.05–0.31) |
| Barbados | 3046.29(2580.35–3526.02) | 622.58(529.88–717.6) | -21.55(-30.45–-11.23) | -1.88(-2.03–-1.73) | 1828.76(1542.15–2137.23) | 825.08(631.51–1040.66) | -23.9(-34.11–-13.18) | 0.17(0.03–0.31) | 1217.54(1005.9–1431.11) | 565.73(469.92–663.88) | -18.34(-30.48–-4.19) | -2.56(-2.71–-2.4) |
| Barbuda | 519.93(454.28–588.68) | 573.39(500.52–648.75) | -21.26(-30.79–-11) | -1.44(-1.6–-1.28) | 316.78(274.33–360.42) | 661.07(558.07–771.49) | -19.53(-30.27–-7.57) | -1.4(-1.55–-1.25) | 203.15(172.07–235.9) | 493.08(420.65–573) | -25.41(-36.4–-11.96) | -2.07(-2.24–-1.91) |
| Belarus | 214423.65(168401.53–267692.65) | 1327.62(1043.35–1656.11) | 5.04(-8.42–19.19) | -1.29(-1.39–-1.2) | 121981.32(95843.73–151291.66) | 1126.51(883.91–1403.17) | -1.31(-15.03–11.96) | -1.62(-1.73–-1.52) | 92442.32(70050.35–120451.39) | 1621.22(1244.13–2077.23) | 15.81(-1.68–35.55) | -0.91(-1–-0.82) |
| Belgium | 76880.56(68356.62–84691.87) | 295.09(263.45–325.85) | -39.42(-44.29–-34.74) | -3.53(-3.72–-3.34) | 44891.08(38619.63–50324.08) | 285.25(249.87–320.63) | -41.8(-47.01–-36.17) | -3.3(-3.5–-3.1) | 31989.48(28757.59–35429.41) | 304.33(273.52–336.96) | -36.96(-42.8–-30.03) | -3.89(-4.07–-3.7) |
| Belize | 1110.61(963.97–1262.75) | 436.49(378.81–494.98) | 11.21(-4.23–28.09) | -1.03(-1.21–-0.85) | 529.85(461.55–598.54) | 420.63(365.24–473.84) | 2.28(-9.51–17.27) | -1.27(-1.45–-1.09) | 580.76(494.59–674.14) | 450.53(383.83–519.54) | 24.46(0.67–51.05) | -0.76(-0.94–-0.57) |
| Benin | 33549.46(27921.16–40748.22) | 732.77(615.46–880.51) | 43.36(18.06–70.17) | -0.75(-0.89–-0.6) | 18831.49(15475.32–22918.69) | 751.98(620.03–912.3) | 51.7(20.45–83.49) | -0.77(-0.91–-0.62) | 14717.97(11549.56–18615.59) | 708.26(563.28–887.12) | 34.47(6.11–70.45) | -0.76(-0.91–-0.62) |
| Bermuda | 443.67(377.22–520.21) | 330.96(283.21–388.83) | 0.45(-11–13.67) | -2.92(-3.1–-2.73) | 210.47(172.47–252.23) | 265.59(219.76–319.1) | -12.4(-24.48–2.24) | -3.63(-3.83–-3.43) | 233.2(197.91–271.6) | 414.92(353.33–480.01) | 15.17(-1.68–36.71) | -2.15(-2.32–-1.97) |
| Bhutan | 2829.17(2293.31–3476.84) | 553.59(450.89–680.02) | 331.15(216.59–459.49) | -0.48(-0.65–-0.31) | 1346.21(1004.67–1696.69) | 537.18(395.87–676.77) | 291.86(181.15–443.6) | -0.72(-0.89–-0.55) | 1482.96(1087.71–1969.54) | 569.79(421.1–755.19) | 377.85(230.54–550.17) | -0.26(-0.43–-0.09) |
| Bolivia (Plurinational State of) | 40193(30342.66–52411.5) | 506.16(385.68–658.18) | 115.21(73.21–167.11) | -1.27(-1.44–-1.09) | 21769(16630.59–28337.54) | 516.69(393.1–671) | 110.84(61.47–168.29) | -1.22(-1.39–-1.04) | 18424(12417.14–26423.52) | 494.33(333.82–704.17) | 119.31(69.37–202.49) | -1.35(-1.52–-1.17) |
| Bosnia and Herzegovina | 103407.53(85567.32–126640.22) | 1775.14(1473.48–2154.13) | 55.1(39.76–69.26) | -0.6(-0.69–-0.51) | 57593.17(47765.75–69792.67) | 1703.94(1413.5–2057.77) | 44.49(27.48–61.96) | -0.79(-0.88–-0.7) | 45814.35(37010.41–58967) | 1838.13(1499.25–2357.09) | 65.97(46.65–86.5) | -0.35(-0.44–-0.26) |
| Botswana | 13608.64(10399.14–17563.46) | 1240.03(956.2–1586.74) | 16.16(-7.64–46.75) | -0.49(-0.6–-0.38) | 7398.3(5524.68–9763.5) | 1152.43(880.72–1510.49) | 20.6(-9.99–63.68) | -0.24(-0.36–-0.12) | 6210.34(4309.63–8099.69) | 1349.08(969.94–1718.7) | 11.29(-16.24–47.3) | -0.73(-0.83–-0.63) |
| Brazil | 1268106.52(1157551.79–1356041.65) | 560.96(510.4–599.82) | 9.34(0.5–18.84) | -3.08(-3.23–-2.94) | 589102.27(525582.83–644593.64) | 460.42(410.77–503.86) | 9.79(-1.82–21.63) | -3.2(-3.35–-3.04) | 679004.25(622339.77–725769.41) | 686.46(628.99–734.52) | 8.84(0.16–19.07) | -2.93(-3.06–-2.8) |
| Brunei Darussalam | 1577.47(1371.19–1811.27) | 735.44(652.96–835.92) | -10.91(-20.64–5.6) | -2.6(-2.73–-2.48) | 795.81(675.21–919.12) | 662.39(576.58–753.04) | -11.85(-23.01–2.28) | -2.55(-2.68–-2.42) | 781.66(667.87–914.73) | 886.84(765.91–1023.55) | -11.22(-25.57–12.49) | -2.45(-2.57–-2.33) |
| Bulgaria | 318646.68(264891.14–377995.77) | 2105.7(1753.31–2502.89) | 40.29(29.86–52.1) | -0.21(-0.3–-0.12) | 171541.73(141565.36–203288.81) | 1855.05(1534.79–2191.74) | 40.86(29–53.54) | -0.42(-0.52–-0.33) | 147104.95(121209.25–176845.28) | 2411.19(1997.4–2889.29) | 37.14(24.53–53.21) | 0.04(-0.04–0.13) |
| Burkina Faso | 43065.05(34537.32–54066.88) | 486.18(391.66–603.02) | 69.37(33.69–109.95) | 0.1(-0.09–0.29) | 23243.14(18050.08–31099.46) | 471.28(373.57–615.28) | 75.05(36.94–125.32) | 0.06(-0.13–0.25) | 19821.91(14967.89–27416.31) | 505.83(384.08–687.49) | 63.67(21.15–111.21) | 0.16(-0.03–0.34) |
| Burundi | 29603.19(23716.42–37583.37) | 759.44(607.13–973.64) | 58.09(24.54–100.95) | -0.94(-1.09–-0.8) | 15437.61(12276.17–19571.88) | 783.35(610.26–1008.35) | 60.37(25.2–103.03) | -0.87(-1.01–-0.73) | 14165.58(10503.81–19775.62) | 731.36(538.91–1035.63) | 58.11(11.54–140.76) | -1.04(-1.19–-0.9) |
| Cabo Verde | 2407.63(2020.95–2913.64) | 566.26(472.33–691.94) | 137.61(93.64–191.5) | 0.61(0.41–0.8) | 1187.48(963.74–1486.65) | 473.7(383.98–591.35) | 140.41(91.08–203.42) | 0.61(0.4–0.81) | 1220.15(999.63–1488.24) | 700.57(573.45–853.39) | 140.53(86.76–203.08) | 0.55(0.37–0.73) |
| Cambodia | 102759.43(84531.72–121155.95) | 1034.14(855.22–1206.47) | 269.42(199.84–343.49) | -0.24(-0.37–-0.11) | 57851.99(48855.1–67497.33) | 981.89(821.74–1144.65) | 280.37(198.74–370.18) | -0.36(-0.49–-0.23) | 44907.45(33043.28–57187.46) | 1109.53(819.44–1402.03) | 255.24(160.82–372.91) | -0.05(-0.18–0.07) |
| Cameroon | 72354.03(56213.76–94234.21) | 655.22(511.02–829.77) | 73.53(36.47–121.12) | 0.16(0–0.32) | 37604.46(28368.96–50924.64) | 645.33(481.69–856.12) | 62.9(27.31–113.09) | -0.08(-0.23–0.08) | 34749.57(24334.21–47262.12) | 666.59(482.32–882.64) | 86.3(34.77–150.27) | 0.41(0.26–0.57) |
| Canada | 201549.92(172578.71–229415.15) | 281.52(239.04–322.89) | -19.76(-25.39–-14.13) | -3.03(-3.24–-2.82) | 109743.26(93096.26–124965.92) | 265.89(224.7–307.14) | -23.02(-29.06–-16.66) | -2.8(-3.01–-2.58) | 91806.67(77127.62–106234.2) | 297.43(249.57–345.53) | -16.8(-24.01–-9.49) | -3.33(-3.53–-3.13) |
| Central African Republic | 17900.06(13196.55–25089.61) | 1021.87(721.55–1426.59) | 31.3(10.9–57.38) | 0.16(0.03–0.29) | 9719.75(6659.08–14114.29) | 989.55(648.49–1453.63) | 44.57(20.34–75.32) | 0.31(0.18–0.44) | 8180.31(5528.17–13079.51) | 1041.35(719.58–1612.84) | 18.81(-7.93–54.19) | -0.04(-0.17–0.09) |
| Chad | 37794.37(29831.31–49329.47) | 673.88(533.37–880.79) | 16.58(-5.95–44.75) | -0.17(-0.33–-0.02) | 18897.86(15022.08–23680.03) | 707.91(554.96–887.13) | 7.8(-15.7–37.8) | -0.15(-0.3–0.01) | 18896.52(13710.51–26679.45) | 645.57(463.24–921.82) | 27.94(-3.28–68.28) | -0.18(-0.33–-0.02) |
| Chile | 100662.75(90439.53–109989.87) | 422.65(379.33–462.44) | 3.06(-7.1–13.25) | -2.22(-2.4–-2.05) | 51479.95(45481.13–56839.32) | 378.97(334.74–419.25) | -1.61(-12.62–8.7) | -2.26(-2.45–-2.08) | 49182.79(44074.14–54193.19) | 476(427.24–523.72) | 7.28(-4.65–20.36) | -2.2(-2.36–-2.04) |
| China | 21393856.76(18720952.02–24375890.76) | 1147.93(1008.58–1302.75) | 157.62(111.73–185.71) | -0.07(-0.19–0.05) | 9599334.1(7942922.05–11345429.1) | 965.78(799.92–1135.34) | 147.59(113.32–183.11) | -0.41(-0.53–-0.28) | 11794522.66(9803614.84–14020975.2) | 1386.35(1161.81–1624.31) | 167.3(102.3–204.86) | 0.29(0.17–0.4) |
| Colombia | 137162.16(110328.12–166980.22) | 256.89(206.68–313.39) | 10.14(-4.26–25.71) | -3.82(-4.02–-3.62) | 74336.87(60401.69–90241.2) | 250.72(203.88–303.82) | 2.18(-12.19–17.21) | -3.98(-4.19–-3.78) | 62825.29(49758.12–78586.51) | 264.36(209.07–331.24) | 15.41(-0.29–32.84) | -3.63(-3.83–-3.43) |
| Comoros | 3759.27(3033.79–4642.76) | 834(667.53–1020.87) | 156.44(104.04–243.6) | -0.6(-0.74–-0.46) | 2366.52(1904.58–2865.62) | 939.92(755.3–1134.84) | 164.65(107.28–249.45) | -0.54(-0.67–-0.41) | 1392.75(1004.11–1985.47) | 702.26(513.68–999.23) | 138.43(74.35–248.96) | -0.75(-0.9–-0.6) |
| Cook Islands | 141.21(119–164.8) | 595.05(500.64–694.1) | 54.02(28.64–81.73) | -1.08(-1.24–-0.92) | 70.62(58.71–84.1) | 1111.46(870.27–1403.18) | 57.61(35.23–83.4) | -0.3(-0.42–-0.18) | 70.59(58.18–82.49) | 613.36(507.36–725.31) | 49.41(11.94–93.18) | -1.13(-1.29–-0.97) |
| Costa Rica | 13293.55(10698.76–16048.76) | 264.01(213.09–319.54) | 5.26(-6.9–19.44) | -2.3(-2.53–-2.07) | 7086.46(5759.48–8464.99) | 574.71(478.13–684.81) | 8.9(-4.21–26.59) | -0.99(-1.15–-0.82) | 6207.09(4965.07–7691.4) | 275.06(219.81–341.04) | 1.29(-12.63–15.85) | -2.28(-2.5–-2.05) |
| Croatia | 82120.22(68222.2–97371.94) | 858.55(714.84–1015.07) | -4.38(-11.7–2.5) | -2.57(-2.68–-2.45) | 46850.06(38636.99–55279.24) | 254.21(206.76–303.84) | -9.81(-17.54–-2.34) | -2.32(-2.55–-2.09) | 35270.17(29067.28–42343.55) | 946.15(784.45–1130.56) | 1.14(-7.66–9.95) | -2.64(-2.75–-2.54) |
| Cuba | 109449.78(91868.65–130018.92) | 564.33(471.31–670.38) | 32.46(18.26–48.18) | -0.66(-0.82–-0.49) | 55127.38(46430.54–65039.17) | 783.59(651.07–925.35) | 29.63(13.74–45.01) | -2.59(-2.71–-2.47) | 54322.4(44861.81–65038.21) | 611.61(506.89–731.26) | 35.57(17.98–55.39) | -0.24(-0.41–-0.08) |
| Cyprus | 6700.05(5786–8056.53) | 380.74(326.34–459.9) | -30.23(-40.99–-16.85) | -3.84(-4–-3.68) | 3870.65(3267.85–4655.61) | 520.14(438.53–614.33) | -32.48(-44.26–-18.84) | -1.06(-1.23–-0.9) | 2829.39(2396.13–3433.88) | 343.06(291.52–412.42) | -26.94(-42.97–-3.06) | -3.43(-3.59–-3.27) |
| Czechia | 139674.41(119040.69–162174.85) | 642.35(546.9–743.21) | -48.32(-52.01–-44.25) | -4.64(-4.75–-4.52) | 78332.79(66542.74–90578.11) | 397.67(338.03–477.25) | -50.07(-54.19–-46.07) | -4.31(-4.47–-4.15) | 61341.62(51571.79–72624.62) | 689.07(581.35–809.83) | -46.8(-51.17–-41.65) | -4.85(-4.96–-4.74) |
| Democratic People's Republic of Korea | 431189.86(363599.69–506658.85) | 1387.08(1172.42–1626.65) | 130(92.9–173.34) | -0.05(-0.16–0.06) | 234933.92(195759.58–283196.8) | 598.09(508.41–691.88) | 122.52(88.43–166.37) | -4.5(-4.62–-4.37) | 196255.94(156720.44–237786.18) | 1631.72(1312.44–1943.48) | 141.17(94.75–203.75) | 0.05(-0.05–0.15) |
| Democratic Republic of the Congo | 230130.46(174755.34–299234.44) | 778.8(577.25–1025.49) | 106.35(71.17–151.77) | -0.42(-0.56–-0.28) | 133650(99698.19–172473.32) | 673.39(548.42–836.08) | 131.96(83.61–192.48) | -0.52(-0.67–-0.37) | 96480.46(69890.17–138827.06) | 771.31(560.09–1102.13) | 79.62(38.47–130.46) | -0.47(-0.61–-0.33) |
| Denmark | 41936.67(37367.27–45984.33) | 341.53(303.7–374.79) | -31.23(-37.69–-24.99) | -3.12(-3.3–-2.94) | 21464.59(18579.55–24008.08) | 1226.39(1028.23–1475.58) | -35.4(-41.97–-28.33) | -0.18(-0.29–-0.07) | 20472.07(18206.44–22678.69) | 384.63(343.14–425.67) | -26.5(-33.88–-17.58) | -3.27(-3.44–-3.11) |
| Djibouti | 4564.3(3545.7–5961.93) | 945.08(754.64–1199.23) | 193.64(129.85–276.92) | 0.24(0.1–0.37) | 2213.18(1737.85–2829.41) | 782.4(567.54–1033.4) | 163.59(100.47–238.5) | -0.41(-0.55–-0.27) | 2351.12(1700.1–3496.46) | 918.44(675.58–1320.38) | 227.33(125.56–364.49) | 0.34(0.2–0.48) |
| Dominica | 607.76(516.13–707.25) | 666.38(566.32–774.19) | 4.63(-7.56–18.83) | -0.09(-0.25–0.07) | 356.17(300.85–420.69) | 303.33(262.99–340.04) | 4.73(-9.01–21.24) | -3.04(-3.23–-2.85) | 251.59(205.21–306.97) | 597.73(490.77–727.92) | 11.36(-9.29–36.13) | -0.34(-0.51–-0.17) |
| Dominican Republic | 67521.03(53633.75–85909.33) | 755.26(603.14–957.93) | 157.08(116.14–210.44) | 1.22(1.06–1.38) | 32915.39(26514.3–42373.73) | 968.09(773.05–1215.87) | 171.78(125.97–236.1) | 0.15(0.01–0.28) | 34605.64(26243.58–45271.32) | 811.51(618.85–1056.56) | 146.14(95.8–201.6) | 1.34(1.19–1.5) |
| Ecuador | 54754.99(44811.95–66654.93) | 385.6(317.67–469.72) | 40.12(19.28–66.07) | -1.29(-1.49–-1.1) | 27702.35(22899.56–33305.89) | 713.6(605.53–841.6) | 41.72(18.22–68.46) | 0.09(-0.06–0.25) | 27052.64(21626.24–33831.83) | 401.41(320.6–500.51) | 38.38(10.59–75.63) | -1.19(-1.38–-1) |
| Egypt | 863082.66(669024.33–1115487.29) | 1381.53(1070.23–1804.15) | 116.44(66.91–180.03) | 0.99(0.88–1.11) | 424529.6(319244.71–557432.57) | 700.28(564.31–896.63) | 138.53(67.67–227.52) | 1.06(0.89–1.23) | 438553.06(322157.31–609900.42) | 1240.62(914.06–1722.83) | 98.36(45.39–170.15) | 0.61(0.49–0.73) |
| El Salvador | 17579.13(13953.06–21750.45) | 283.8(224.14–353.4) | 55.95(24.13–91.9) | -2.03(-2.26–-1.81) | 9685.82(7575.43–12037.85) | 370.38(306.29–443.04) | 64.63(36.53–101.33) | -1.43(-1.62–-1.23) | 7893.31(6096.17–10197.88) | 304.27(235.33–394.2) | 44.61(10.73–113.35) | -2.18(-2.39–-1.96) |
| Equatorial Guinea | 3311.69(2577.98–4325.13) | 802.04(622.28–1044.12) | 92.5(32.66–151.64) | -0.91(-1.05–-0.77) | 2003.5(1496.05–2776.82) | 1617.91(1201.93–2121.11) | 112.55(34.93–215.1) | 1.57(1.46–1.68) | 1308.19(970.54–1808.12) | 761.34(555.83–1048.02) | 66.5(17.34–137.17) | -1.66(-1.8–-1.52) |
| Eritrea | 17671(14084.96–22918.95) | 826.11(656.3–1063.36) | 281.41(191.72–390.34) | 0.29(0.15–0.44) | 10948.41(8460.38–14240.18) | 268.67(208.69–335.54) | 290.47(178.16–473.45) | -1.85(-2.08–-1.61) | 6722.58(4649.83–9310.54) | 758.16(524.33–1037.04) | 258.15(165.75–423.61) | -0.14(-0.29–0.01) |
| Estonia | 14956.44(12160.91–18942.87) | 536.88(440.4–671.73) | -54.42(-58.93–-43.65) | -5.87(-5.98–-5.76) | 8836.6(7116.02–11415.73) | 832.47(621.09–1126.54) | -59.1(-63.98–-48.23) | -0.37(-0.51–-0.23) | 6119.84(4899.72–7589.89) | 630.13(506.04–782.3) | -48.63(-54.09–-38.19) | -5.66(-5.76–-5.56) |
| Eswatini | 5421.58(4226.7–6892.11) | 1146.66(896.52–1453.85) | 21.67(0.49–50.75) | 0.19(0.07–0.31) | 3171.44(2318.01–4230.27) | 858.29(656.17–1118.3) | 26.58(-0.17–63.43) | 0.56(0.41–0.71) | 2250.13(1735.38–2848.04) | 1236.01(980.94–1529.3) | 16.77(-7.75–50.88) | 0.21(0.09–0.32) |
| Ethiopia | 187549.72(156926.24–229109.98) | 494.07(407.21–605.33) | 222.44(115.52–349.02) | -0.5(-0.68–-0.32) | 101130.45(84577.77–125080.62) | 472.82(385.73–596.75) | 247.48(142.21–368.22) | -6.07(-6.19–-5.95) | 86419.27(65516.99–117514.66) | 457.79(339.69–630.81) | 196.82(71.66–426.17) | -0.71(-0.9–-0.53) |
| Fiji | 6109.93(5037.94–7330.9) | 943.09(786.38–1114.81) | 34.79(16.99–59.75) | -0.76(-0.89–-0.64) | 3169.35(2586.01–3786.01) | 1072.26(777.83–1423.08) | 47.27(22.41–78.55) | 0.18(0.06–0.3) | 2940.58(2349.08–3603.45) | 999.58(811.42–1202.22) | 23.32(0.27–51.81) | -1.11(-1.23–-0.99) |
| Finland | 53683.45(47515.55–59155.77) | 394.92(350.36–436.48) | -24.42(-30.84–-18.27) | -3.42(-3.59–-3.26) | 30399(25975.86–34094.4) | 532.51(446.96–653.89) | -29.88(-37.11–-23.11) | -0.26(-0.44–-0.08) | 23284.44(20935.41–25520.9) | 420.7(378–461.97) | -17.08(-25.32–-8.65) | -3.65(-3.8–-3.49) |
| France | 376572.92(325695.69–419131.34) | 236.54(207.38–264.8) | -29.39(-35.2–-23.53) | -3.16(-3.38–-2.94) | 211326.45(176182.59–238936.39) | 907.73(749.1–1082.91) | -33.18(-39.47–-27.53) | -0.36(-0.5–-0.23) | 165246.47(146930.88–183167.8) | 266.01(236.12–294.93) | -25.87(-33.02–-18.28) | -3.53(-3.73–-3.33) |
| Gabon | 7957.33(6545.38–9468.73) | 893.95(741.9–1053.17) | 61.39(32.94–97.09) | -0.28(-0.42–-0.15) | 4246.66(3431.61–5192.57) | 368.28(318.37–415.6) | 48.7(16.77–86.86) | -3.3(-3.48–-3.13) | 3710.67(2906.89–4646.85) | 946.26(748.42–1170.37) | 74.83(32.18–127.43) | -0.26(-0.39–-0.13) |
| Gambia | 7294.01(5935.28–8812.56) | 784.52(650.73–936.48) | 150.17(94.59–216.08) | 0.05(-0.1–0.2) | 3995.19(3218.03–4876.32) | 212.09(181.3–241.52) | 154.83(96.13–229.06) | -2.92(-3.15–-2.68) | 3298.82(2574.16–4220.12) | 750.02(592.33–944.09) | 141.49(76.38–230.91) | -0.05(-0.2–0.1) |
| Georgia | 74654.99(63817.75–86559.59) | 1189.28(1020.09–1371.68) | 35.82(18.35–58.65) | -0.46(-0.58–-0.34) | 39640.48(33650.01–46063.66) | 841.73(675.16–1033.21) | 21.83(2.89–44.43) | -0.37(-0.51–-0.24) | 35014.52(29538.78–40737.29) | 1495.65(1265.03–1740.49) | 58.19(36.04–90.45) | 0.1(-0.01–0.21) |
| Germany | 743272.96(658532.43–817759.67) | 350.72(311.52–385.93) | -39.85(-45.01–-34.69) | -3.87(-4.05–-3.7) | 410444.41(357819.95–454610.59) | 811.01(652.44–995.13) | -44.21(-49.27–-38.71) | 0.13(-0.02–0.28) | 332828.55(297635.03–366398.19) | 378.45(339.57–416.27) | -32.93(-39.43–-25.54) | -4.26(-4.43–-4.1) |
| Ghana | 168541.38(141291.25–201135.65) | 1165.89(985.2–1374.31) | 123.44(82.74–167.89) | 0.31(0.19–0.44) | 109167.24(89608.74–131531.89) | 990.46(845.34–1148.92) | 115.89(75.8–163.52) | -0.99(-1.11–-0.86) | 59374.14(47858.64–72414.99) | 926.27(756.81–1120.99) | 139.64(83.75–204.07) | 0.78(0.64–0.92) |
| Greece | 151349.01(133366.72–164482.35) | 492.28(439.35–534.54) | -24.26(-30.49–-18.36) | -3.73(-3.87–-3.58) | 91454.78(79193.5–99913.68) | 323.66(283.55–362.17) | -22.69(-28.87–-17.02) | -3.74(-3.93–-3.55) | 59894.23(53002.89–65802.85) | 465.83(415.13–510.31) | -27.26(-34.62–-19.14) | -3.79(-3.93–-3.64) |
| Greenland | 400.33(339.22–466.06) | 710.97(606.45–823.73) | 16.74(4.53–30.55) | -3.3(-3.42–-3.17) | 157.95(128.09–189.97) | 1333.77(1104.48–1602.47) | -4.93(-16.26–8.69) | -0.07(-0.18–0.04) | 242.38(203.42–283.15) | 835.17(706.64–963.02) | 37.35(17.12–59.49) | -2.41(-2.53–-2.29) |
| Grenada | 904.36(810.06–996.09) | 904.08(805.74–994.44) | -31.47(-40.03–-20.3) | -1.9(-2.02–-1.78) | 512.53(452.32–574.29) | 511.09(450.92–557.47) | -29.58(-38.56–-16.08) | -3.64(-3.78–-3.5) | 391.83(348.03–440.75) | 865.58(770.62–969.29) | -32.21(-43.65–-16.13) | -1.86(-1.98–-1.75) |
| Grenadines | 971.02(851.92–1095.46) | 763.46(669.83–857.66) | -11.35(-20.62–-0.96) | -1.74(-1.87–-1.6) | 550.35(482.75–621.63) | 579.29(474.09–690.83) | -10.84(-20.62–-0.48) | -4.13(-4.26–-4) | 420.67(362.35–482.12) | 659.2(568.09–749.57) | -7.27(-21.59–9.12) | -1.81(-1.95–-1.66) |
| Guam | 1095.79(924.64–1274.25) | 597.89(504.25–694.32) | 33.53(19.09–49.22) | -1.41(-1.57–-1.25) | 570.39(475.31–666.39) | 910.16(805.25–1016.66) | 37.55(21.6–54.97) | -1.92(-2.05–-1.8) | 525.4(439.91–621.51) | 600.91(503.12–707.02) | 28.15(8.27–52.25) | -1.36(-1.52–-1.21) |
| Guatemala | 34557.3(28379.41–41478.69) | 331.99(275.19–396.59) | 119.86(80.41–182.39) | -1.9(-2.1–-1.7) | 18916.4(15522.94–22807.78) | 593.51(493.92–693.95) | 126.28(79.79–205.04) | -1.43(-1.58–-1.27) | 15640.9(12820.05–18899.49) | 353.68(292.37–422.8) | 112.92(67.81–186.31) | -1.36(-1.56–-1.17) |
| Guinea | 37760.71(30416.03–46339.65) | 696.69(566.63–850.41) | 74.72(49.45–105.56) | 0.3(0.14–0.45) | 20378.59(16250.85–25642.06) | 322.09(264.13–388.31) | 64.62(31.71–107.91) | -2.27(-2.47–-2.07) | 17382.12(13008.67–22557.69) | 647.15(488.94–834.24) | 90.18(51.74–142.87) | 0.68(0.51–0.84) |
| Guinea-Bissau | 6036.27(4909.92–7455.19) | 883.42(722.37–1076.18) | 110.65(67.44–171.64) | -0.02(-0.15–0.12) | 3361.99(2654.78–4279.09) | 746.63(597.18–953.31) | 122.08(72.6–189.37) | -0.02(-0.18–0.13) | 2674.27(2074.47–3368.7) | 871.31(685.44–1086.77) | 96.49(39.89–195.12) | -0.11(-0.24–0.03) |
| Guyana | 7348.64(5867.71–9044.98) | 1335.65(1073.5–1636.15) | 9.29(-4.84–26.2) | -1.58(-1.69–-1.48) | 3665.75(2945.62–4487.47) | 889.12(700.95–1122.64) | 17.64(0.75–37.34) | 0.05(-0.09–0.18) | 3682.89(2842.94–4674.45) | 1422.63(1113.53–1785.33) | 2.07(-14.67–24.53) | -1.75(-1.85–-1.65) |
| Haiti | 76530.46(54374.56–115642.88) | 1263.05(884.7–1892.39) | 65.13(32.99–104.81) | -0.38(-0.5–-0.27) | 48219.82(32048.59–71946.48) | 1250.46(1012.58–1524.62) | 75.03(38.8–124.11) | -1.39(-1.5–-1.28) | 28310.65(18904.67–47303.22) | 997.76(659.37–1676.78) | 49.2(6.95–105.21) | -0.84(-0.97–-0.72) |
| Honduras | 46570.51(38718.4–58891.07) | 851.67(710.11–1096.07) | 152.38(102.28–225.88) | 1(0.85–1.15) | 23736.18(18964.96–30128.05) | 1499.73(986.33–2199.28) | 141.75(90.79–229.12) | -0.06(-0.17–0.04) | 22834.33(17762.08–30991.5) | 888.9(689.37–1209.55) | 162.39(96.45–248.54) | 1.08(0.93–1.23) |
| Hungary | 172558.96(146569.04–200189.09) | 870.14(736.5–1009.57) | -25.07(-29.79–-20.39) | -3.25(-3.36–-3.14) | 96339.64(81315.91–111409.98) | 818.78(666.07–1037.69) | -28.09(-33.58–-22.69) | 0.93(0.78–1.08) | 76219.31(64592.37–89342.15) | 1001.08(846.95–1173.56) | -23.52(-28.86–-17.32) | -3.2(-3.3–-3.1) |
| Iceland | 1402.81(1210.35–1584.1) | 231.37(200.44–261.13) | -34.6(-40.11–-28.77) | -3.66(-3.86–-3.45) | 702.22(583.42–807.4) | 768.64(651.27–885.12) | -35.98(-42.17–-28.96) | -3.26(-3.38–-3.14) | 700.59(616.01–784.74) | 263.9(232.54–295.61) | -33.08(-39.8–-25.94) | -3.59(-3.79–-3.4) |
| India | 5689263.86(4821067.25–6649548.06) | 541.35(461.58–633.24) | 153.54(113.07–195.79) | -1.17(-1.34–-1.01) | 2680175.56(2154767.91–3217503.02) | 201.56(171.22–231.45) | 184.81(127.16–260.6) | -3.73(-3.95–-3.51) | 3009088.31(2320914.85–3853520.11) | 594.59(466.95–753.58) | 130.18(90.39–177.13) | -1.14(-1.3–-0.99) |
| Indonesia | 2957904.29(2298331.42–3475193.84) | 1654.43(1322.3–1917.55) | 209.05(171.04–246.89) | 1.16(1.05–1.27) | 1508303.28(1189954–1842793.68) | 492.46(395.31–587.03) | 198.92(160.19–246.54) | -1.15(-1.32–-0.97) | 1449601.01(1027768.23–1902728.37) | 1705.59(1248.75–2180.86) | 221.49(171.95–273.25) | 1.45(1.34–1.56) |
| Iran (Islamic Republic of) | 668904.04(607975.77–717571.29) | 984.37(889.98–1054.69) | 112.82(79.42–144.82) | -2.13(-2.25–-2.02) | 332874.78(298050–363102.64) | 1593.53(1258.77–1919.84) | 110.7(78.98–148.8) | 0.89(0.78–1) | 336029.26(307279.31–362853.14) | 971.06(883.23–1048.77) | 113.38(74.43–152.15) | -2.09(-2.21–-1.98) |
| Iraq | 373158.63(305766.24–438919.33) | 1828.87(1525.31–2123.63) | 93.52(60.37–127.37) | -0.26(-0.36–-0.17) | 181971.24(149901.68–213417.98) | 1001.43(895.88–1091.21) | 84.05(49.38–122.46) | -2.11(-2.22–-2) | 191187.38(152707.3–227196.7) | 1942.26(1576.5–2250.43) | 102.5(59.95–155.86) | -0.21(-0.31–-0.12) |
| Ireland | 21902.81(19019.75–24311.7) | 284.71(247.96–316.81) | -44.24(-49.49–-39.39) | -4.37(-4.55–-4.18) | 11915.8(10186.34–13434.43) | 1720.32(1434.79–1986.43) | -45.67(-51.02–-40.64) | -0.32(-0.42–-0.22) | 9987.01(8732.81–11191.47) | 295.67(258.88–331.27) | -43.16(-49.07–-36.73) | -4.65(-4.83–-4.48) |
| Israel | 29557.38(25830.74–32762.8) | 242.26(211.72–269.65) | -32.35(-39.34–-25.26) | -3.57(-3.78–-3.36) | 16652.23(14241.82–18593.66) | 272.25(233.28–308.23) | -29.13(-37.02–-20.64) | -4.12(-4.31–-3.92) | 12905.15(11379.62–14443.73) | 243.07(214.55–272.08) | -36.24(-44.38–-26.48) | -3.73(-3.93–-3.52) |
| Italy | 527796.25(456052.75–577588.23) | 298.89(261.6–328.24) | -32.7(-38.72–-28.84) | -4.18(-4.37–-3.99) | 313705.8(264875.34–349287.36) | 239.34(204.69–268.3) | -31.62(-38.98–-27) | -3.47(-3.68–-3.26) | 214090.44(192309.32–231292.84) | 313.9(280.79–340.5) | -35.48(-40.09–-31.86) | -4.52(-4.7–-4.34) |
| Ivory Coast | 67593.85(53759.78–84247.3) | 690.58(569.7–838.72) | 79.48(45.94–113.7) | -0.81(-0.95–-0.66) | 32252.79(25961.83–40150.84) | 285.47(243.86–318.91) | 91.4(54.45–133.66) | -3.93(-4.12–-3.73) | 35341.06(26808.99–45958.15) | 708.47(559.41–897.51) | 69.81(32.36–118.09) | -1.02(-1.16–-0.88) |
| Jamaica | 21968.65(18250.31–26014.51) | 719.19(596.97–850.85) | -2.89(-14.98–12.85) | -1.01(-1.16–-0.86) | 12266.79(10136.4–14493.29) | 722.24(596.12–857.17) | -5.1(-18.2–11.61) | -1.35(-1.5–-1.2) | 9701.85(7906.9–11717.95) | 707.79(576.07–854.36) | 0.64(-14.33–22.63) | -0.59(-0.74–-0.44) |
| Japan | 1360713.92(1131346.6–1540307.05) | 326.57(276.64–373.41) | -13.86(-21.26–-8.05) | -3.63(-3.81–-3.45) | 691838.53(552866.81–801992.62) | 266.41(218.17–314.24) | -15.61(-24.82–-8.14) | -3.86(-4.07–-3.66) | 668875.39(583722.28–741333.18) | 398.86(349.88–446.27) | -12.17(-17.63–-7.55) | -3.56(-3.72–-3.4) |
| Jordan | 59926.45(50567.84–69639.2) | 1083.51(911.65–1252.34) | 25.71(9.03–42.32) | -2.59(-2.69–-2.49) | 30630.06(25569.34–35997.6) | 1192.99(991.58–1403.78) | 15.94(-0.87–35.32) | -2.84(-2.94–-2.75) | 29296.39(22698.3–35702.03) | 985.4(748.05–1187.98) | 39.68(14.47–67.62) | -2.16(-2.27–-2.05) |
| Kazakhstan | 260682.49(229951.36–294014.46) | 1715.39(1515.35–1929.68) | 17.27(-2.14–33.95) | -1.03(-1.12–-0.94) | 138889.24(122430.12–156985.71) | 1489.44(1317.89–1682.45) | 6.44(-11.71–22.56) | -1.25(-1.35–-1.16) | 121793.24(105687.22–139273.24) | 2054.46(1783.89–2342.96) | 32.12(8.5–55.77) | -0.81(-0.89–-0.73) |
| Kenya | 132481.86(109993.14–159069.11) | 703.21(572.35–838.61) | 105.13(85–131.37) | 0.31(0.15–0.47) | 72557.05(58574.8–89994.08) | 688.99(552.48–846.16) | 103.19(80.52–130.39) | 0.03(-0.13–0.19) | 59924.81(47973.58–79746.41) | 719.93(562.6–954.1) | 108.69(77.56–149.65) | 0.61(0.45–0.77) |
| Kiribati | 905.8(740.63–1083.32) | 1456.44(1205.8–1722.73) | 47.63(23.31–72.46) | -0.3(-0.41–-0.19) | 396.64(317.06–489.73) | 1149.63(924.51–1412.81) | 56.77(25.24–93.59) | -0.09(-0.21–0.03) | 509.16(402.87–629.05) | 1904.18(1545.2–2290.32) | 41.08(12.13–74.05) | -0.37(-0.47–-0.28) |
| Kuwait | 15225.1(12983.55–17974.04) | 645.42(546.47–754.36) | 88.83(66.14–112.99) | 0.39(0.24–0.55) | 5329.09(4370.12–6344.53) | 530.8(438.37–625.11) | 24.75(8.78–43.49) | -0.76(-0.91–-0.6) | 9896.01(8109.51–11898.03) | 720.74(588.31–865.86) | 151.67(117.47–193.57) | 1.16(1.01–1.32) |
| Kyrgyzstan | 53950.08(47821.13–60948.08) | 1287.44(1142.42–1453.48) | 2.77(-8.24–15.41) | -1.77(-1.87–-1.68) | 24515.95(21783.5–27798.45) | 1034.88(921.17–1169.5) | -18.3(-28.34–-7.56) | -2.27(-2.37–-2.17) | 29434.13(25936.81–33517.37) | 1624.88(1431.85–1836.55) | 31.03(14.62–51.97) | -1.36(-1.44–-1.27) |
| Lao People's Democratic Republic | 46822.22(37699.46–58746.85) | 1267.48(1029.35–1568.04) | 226.48(172.58–291.4) | -0.23(-0.34–-0.12) | 21681.01(17930.49–26388.59) | 1127.78(938.73–1368.17) | 232.14(170.93–315.66) | -0.25(-0.37–-0.13) | 25141.21(18679.6–33574.48) | 1418.74(1077.94–1875.05) | 221.75(150.21–313.05) | -0.23(-0.33–-0.12) |
| Latvia | 59291.52(51025.79–69569.63) | 1361.68(1171.99–1594.86) | 6.61(-2.01–15.41) | -2.25(-2.34–-2.16) | 37437.19(30673.85–45872.24) | 1230.79(1019.73–1506.63) | -0.39(-9.14–7.43) | -2.33(-2.43–-2.23) | 21854.33(17891.9–26868.6) | 1531.26(1256.67–1872.24) | 12.44(2.57–24.59) | -2.25(-2.33–-2.16) |
| Lebanon | 31068.2(24586.52–38150.91) | 597.83(473.25–734.25) | 100.16(57.32–140.76) | -0.23(-0.4–-0.07) | 17599.6(13640.92–21646.34) | 617.14(478.42–759.77) | 79.57(41.56–123.73) | -0.64(-0.8–-0.48) | 13468.6(10071.48–17425.42) | 573.86(430.5–742.13) | 121.42(58.19–184.39) | 0.3(0.13–0.47) |
| Lesotho | 12134.53(9094.87–15852.85) | 1183.58(907.32–1516.69) | 2.13(-15.71–23.9) | 1.45(1.32–1.58) | 7199.84(5092.85–9915.71) | 1138.76(816.91–1547.98) | 4.07(-19.58–34.65) | 1.84(1.7–1.97) | 4934.69(3237.09–6774.75) | 1208.06(830.42–1603.97) | -2.15(-23.44–23.76) | 0.83(0.71–0.95) |
| Liberia | 11745.66(9288.9–14923.79) | 613.11(495.19–776.19) | 96.12(54.56–148) | -0.73(-0.88–-0.57) | 6442.73(5114.46–8160.09) | 673.82(532.37–853.75) | 91.96(47.42–147.84) | -0.78(-0.93–-0.63) | 5302.92(3994.54–7539.88) | 552.8(417.03–761.14) | 98.49(45.17–167.82) | -0.73(-0.9–-0.56) |
| Libya | 55357.25(43125.84–71016.24) | 1122.57(875.9–1435.2) | 108.59(71.46–157.74) | 0.65(0.52–0.78) | 30355.19(23281.84–38071.41) | 1244.79(963.12–1555.49) | 114.14(76.91–158.99) | 0.73(0.61–0.86) | 25002.05(18343.46–35863.8) | 1004.46(735.76–1436.14) | 100.73(46.44–174.62) | 0.51(0.38–0.65) |
| Lithuania | 62344.86(53382.53–73069.98) | 1012.83(870.52–1186.41) | 27.19(19.47–35.38) | -1.21(-1.32–-1.1) | 37730.35(31864.21–44148.09) | 895.99(761.73–1050.4) | 19.99(11.12–28.71) | -1.52(-1.63–-1.4) | 24614.5(20682.65–29232.93) | 1175.53(988.58–1391.64) | 31.9(21.32–43.55) | -0.84(-0.95–-0.74) |
| Luxembourg | 3009.76(2589.29–3401.05) | 278.47(240.03–314.84) | -59.91(-63.81–-56.36) | -5.2(-5.37–-5.02) | 1664.66(1399.25–1899.33) | 257.35(217.38–294.39) | -63.04(-67.02–-59.05) | -5(-5.18–-4.82) | 1345.1(1155.01–1534.01) | 302.93(260.11–344.23) | -56.23(-60.89–-51.35) | -5.5(-5.67–-5.34) |
| Madagascar | 103036.46(81074.67–132267.83) | 1113.12(877.89–1410.52) | 114.87(75.74–161.82) | 0.36(0.24–0.49) | 59534.7(46820.32–77737.85) | 1228.23(951.62–1576.45) | 130.87(87.27–180.57) | 0.4(0.28–0.52) | 43501.76(31548.85–62446.07) | 979.26(720.09–1378.48) | 95.23(44.94–171.01) | 0.22(0.09–0.35) |
| Malawi | 56397.09(46263.39–68582) | 859.75(701.81–1045.97) | 190.19(133.83–262.68) | -0.17(-0.31–-0.03) | 29884.99(23949.37–36404.98) | 776.5(619.41–948.9) | 190.34(124.24–265.67) | -0.6(-0.75–-0.46) | 26512.1(20922.57–36285.42) | 976(763.33–1332.99) | 193.56(121.18–294.82) | 0.35(0.22–0.48) |
| Malaysia | 198476.72(162121.5–241254.34) | 811.48(666.68–988.67) | 20.85(5.39–39.02) | -1.01(-1.14–-0.87) | 99311.86(81881–119876.09) | 826.88(679.69–993) | 20.31(2.5–40.66) | -0.94(-1.07–-0.8) | 99164.86(79324.68–123941.96) | 794.85(633.92–987.47) | 22.51(0.12–51.98) | -1.07(-1.22–-0.93) |
| Maldives | 1500.65(1268.36–1752.95) | 544.37(460.58–633.94) | 122.84(80.89–172.13) | -2.51(-2.66–-2.35) | 592.37(490.83–710.6) | 473.34(393.69–567.69) | 154.51(101–222.15) | -2.96(-3.12–-2.81) | 908.28(763.93–1067.04) | 607.71(508.97–718.97) | 100.01(55.96–155.35) | -2.13(-2.28–-1.98) |
| Mali | 51828.32(42679.67–63108.18) | 619.71(514.14–745.95) | 45.2(22.33–73.55) | -0.67(-0.83–-0.51) | 31252.28(24703.63–39410.66) | 740.27(594.99–900.9) | 38.27(9.89–73.33) | -0.87(-1.01–-0.73) | 20576.03(15553.13–27459.06) | 506.42(376.72–671.91) | 61.97(27.07–106.98) | -0.27(-0.45–-0.08) |
| Malta | 3172.84(2740.08–3583.01) | 320.24(277.92–361.08) | -26.48(-33.25–-19.64) | -3.63(-3.81–-3.45) | 1745.1(1461.13–1990.65) | 304.12(259.28–346.58) | -24.65(-33.07–-17.29) | -3.71(-3.89–-3.53) | 1427.74(1248.7–1608.63) | 341.25(297.98–384.05) | -28.55(-36.45–-20.35) | -3.52(-3.7–-3.35) |
| Marshall Islands | 305.28(240.54–392.76) | 1045.2(840.9–1324.05) | 42.53(23.6–62.36) | -0.09(-0.21–0.04) | 157.86(123.07–201.49) | 1130.99(889.42–1454.18) | 42.75(21.26–68.6) | 0.34(0.21–0.46) | 147.42(108.5–202.36) | 968.38(738.18–1330.04) | 40.11(15.49–72.55) | -0.54(-0.67–-0.41) |
| Mauritania | 12302.84(9990.33–15056.15) | 618.26(509.12–745.78) | 54.18(26.13–87.06) | -1.61(-1.76–-1.46) | 7258.2(5845.72–8965.45) | 734.93(590.58–892.69) | 44.71(14.07–83.56) | -1.45(-1.58–-1.31) | 5044.65(3885.09–6528.1) | 505.24(396.17–644.2) | 66.96(26.77–116.26) | -1.69(-1.86–-1.53) |
| Mauritius | 11190.17(9456.11–13023.64) | 697.71(590.64–810.56) | -25.39(-31.19–-19.35) | -3.94(-4.06–-3.82) | 5537.1(4676.24–6459.02) | 616.12(522.25–720.72) | -21.9(-28.66–-14.51) | -3.71(-3.84–-3.58) | 5653.07(4700.39–6656.07) | 796.62(670.55–929.7) | -28.71(-35.72–-20.65) | -4.11(-4.22–-4) |
| Mexico | 380771.74(333787.66–433774.24) | 343.16(301.82–390.28) | 26.3(15.02–37.02) | -2.17(-2.37–-1.98) | 197735.42(169290.94–229853.6) | 331.56(283.26–386.2) | 21.92(9.82–34.8) | -2.33(-2.52–-2.13) | 183036.31(152310.5–216999.17) | 356.32(297.35–421.43) | 30.56(16.98–42.9) | -2.01(-2.21–-1.82) |
| Micronesia (Federated States of) | 627.22(468.41–802.97) | 1056.13(827.09–1300.13) | 39.3(2.94–72.32) | -0.28(-0.4–-0.15) | 321.05(245.33–402.29) | 1036.04(803.78–1279.53) | 50.73(20.68–93.13) | -0.02(-0.15–0.11) | 306.17(194.55–429.08) | 1064.17(733.39–1431.71) | 28.85(-18.49–66.22) | -0.58(-0.7–-0.45) |
| Monaco | 433.61(357.85–517.87) | 396.97(330.42–472.74) | -45.21(-51.61–-29) | -2.78(-2.96–-2.61) | 259.92(205.78–310.15) | 411.55(328.44–489.57) | -46.75(-54.45–-36.25) | -2.49(-2.67–-2.32) | 173.68(138.81–222.74) | 373.58(299.08–469.28) | -42.64(-53.82–2.49) | -3.1(-3.28–-2.92) |
| Mongolia | 12772.05(10292.86–16014.01) | 574.46(466.01–724.96) | 139.57(101.14–179.15) | 0.8(0.63–0.98) | 5999.6(4831.16–7472.74) | 492.33(398.4–614.8) | 124.95(85.1–170.82) | 0.17(0–0.35) | 6772.45(5254.86–9057.45) | 687.02(542.75–922.5) | 164.21(105.15–239.95) | 1.43(1.27–1.6) |
| Montenegro | 6949.23(5917.57–8017.25) | 730.28(623.31–840.23) | 69.17(46.53–90.74) | 1.35(1.19–1.51) | 3723.67(3152.55–4317.99) | 681.9(579.2–789.17) | 64.54(41.11–91) | 1.41(1.24–1.57) | 3225.56(2590.96–3867.69) | 797.85(636.68–952.8) | 73.14(36.71–106.76) | 1.24(1.09–1.4) |
| Morocco | 451673.61(364059.6–544462.07) | 1603.6(1305.05–1921.74) | 197.09(144.34–255.09) | 0.74(0.63–0.84) | 241882.59(189697.75–297703.84) | 1669.66(1328.63–2024.16) | 191.23(135.99–260.12) | 0.87(0.76–0.97) | 209791.02(159589.35–268425.46) | 1536.76(1189.53–1957.9) | 202.84(134.93–291.08) | 0.59(0.48–0.7) |
| Mozambique | 131494.2(105148.35–165179.08) | 1332.51(1071.3–1667.31) | 117.85(69.69–174.42) | 1.34(1.21–1.46) | 62126.09(47914.58–81079.35) | 1107.56(847.34–1460.33) | 103.49(57.7–161.14) | 0.68(0.54–0.81) | 69368.1(52641.73–90656.51) | 1610.94(1261.41–2080.09) | 133.53(65.31–216.22) | 1.98(1.87–2.1) |
| Myanmar | 597175.29(498286.66–700052.41) | 1497.32(1267.13–1733.16) | 188.57(137.47–252.36) | -0.45(-0.55–-0.34) | 302542.62(254565.26–352703.21) | 1333.02(1126.99–1549.82) | 229.16(160.83–314.62) | -0.23(-0.34–-0.12) | 294632.67(230931.65–361833.54) | 1705.96(1361.36–2060.33) | 156.59(103.56–229.2) | -0.58(-0.68–-0.49) |
| Namibia | 13645.96(11316.82–16384.02) | 1105.95(922.78–1326.75) | 19.44(-1.46–44.94) | -0.68(-0.79–-0.56) | 7258.99(5769.12–9031.1) | 976.38(776.71–1215.14) | 21.3(-1.45–57.95) | -0.97(-1.09–-0.85) | 6386.97(5085.86–7959.87) | 1288.45(1011.47–1584.15) | 17.39(-9.67–51.89) | -0.28(-0.4–-0.17) |
| Nauru | 59.95(46.24–77.49) | 1559.07(1255.35–1903.68) | 17(-0.91–37.32) | -0.16(-0.26–-0.06) | 31.46(23.66–41.28) | 1589.45(1255.74–1981.35) | 42.93(13.77–74.6) | 0.36(0.25–0.46) | 28.49(19.01–39.09) | 1546.1(1127.06–2020.7) | -2.63(-21.17–20.24) | -0.51(-0.6–-0.41) |
| Nepal | 114408.32(83374.98–159404.29) | 576.33(424.39–792.11) | 265.22(178.82–377.07) | -0.5(-0.67–-0.33) | 42686.12(31161.58–55968.43) | 416.15(302.57–549.31) | 228.17(132.06–365.95) | -1.09(-1.28–-0.9) | 71722.2(48552.13–111319.18) | 753.69(515.25–1155.71) | 291.69(185.38–439.98) | -0.06(-0.21–0.1) |
| Netherlands | 118812.66(104893.39–131674.75) | 330.29(291.89–366.2) | -24.05(-29.73–-18.21) | -3.01(-3.2–-2.83) | 67147.22(58141.55–75544.38) | 320.43(277.66–358.79) | -28.4(-34.23–-22.5) | -2.76(-2.95–-2.56) | 51665.44(46196.05–57673.42) | 335.1(300.03–372.37) | -19.2(-26.93–-11.06) | -3.42(-3.6–-3.24) |
| New Zealand | 25057.59(21652.49–27871.14) | 301.66(262.59–337.53) | -19.83(-27.23–-12.13) | -3.2(-3.39–-3) | 14449.81(12178.68–16269.08) | 308.89(263.07–349.28) | -22.59(-30.12–-15.55) | -2.83(-3.02–-2.63) | 10607.78(9306.15–11903.49) | 289.41(254.09–324.58) | -17.63(-26.1–-7.69) | -3.67(-3.86–-3.47) |
| Nicaragua | 17921(15272.16–20637.09) | 469.63(402.35–534.07) | 113.39(81.52–154.55) | -1.23(-1.4–-1.06) | 9135.72(7789.19–10476.16) | 424.84(361.32–485.2) | 137.43(103.25–181.82) | -0.86(-1.04–-0.68) | 8785.28(7146.77–10562.85) | 528.33(434.43–629.26) | 93.12(56.48–152.13) | -1.62(-1.78–-1.47) |
| Niger | 48162.99(36710.11–63566.2) | 650.1(492.6–837.53) | 82.37(45.96–125.5) | -0.69(-0.84–-0.53) | 27263.64(20769.82–35209.45) | 709.22(535.75–895.6) | 92.12(49.75–149.39) | -0.86(-1.01–-0.71) | 20899.35(14628.77–30028.8) | 587.69(403.55–849.5) | 71.77(23.19–133.87) | -0.52(-0.69–-0.35) |
| Nigeria | 525144.27(428259.25–646331.86) | 657.98(538.67–794.32) | 38.29(3.5–83.35) | -0.86(-1.01–-0.71) | 295989.18(234810.36–375075.37) | 713.91(558.34–899.32) | 32.23(-4.27–76.55) | -0.89(-1.03–-0.74) | 229155.09(170504.51–316156.51) | 601.42(447.64–821.65) | 44.45(0.19–111.33) | -0.71(-0.87–-0.55) |
| Niue | 18.79(14.74–23.25) | 890.78(698.87–1103.49) | 1.91(-11.59–16.87) | -0.73(-0.86–-0.6) | 9.77(7.65–12.64) | 816.88(638.61–1057.83) | -2.49(-16.84–13.58) | -0.58(-0.72–-0.44) | 9.01(6.81–11.16) | 979.19(737.23–1203.95) | 7.44(-13.37–30.98) | -0.88(-1.01–-0.76) |
| North Macedonia | 80513.75(67161.35–95299.21) | 2856.22(2411.17–3340.97) | 44.4(31.98–55.98) | -0.43(-0.51–-0.36) | 43214.45(36221.3–51003.46) | 2820.67(2365.22–3313.63) | 47.79(33.92–63.29) | -0.43(-0.51–-0.36) | 37299.3(30663.18–44979.35) | 2871.65(2405.85–3405.95) | 40.68(24.22–55.95) | -0.46(-0.53–-0.39) |
| Northern Mariana Islands | 283.99(242.29–330.18) | 677.99(584.13–772.91) | 42.7(22.84–66.27) | -1.76(-1.9–-1.62) | 133.67(111.7–157.85) | 659.77(553.55–775.42) | 41.84(21.5–64.54) | -2.25(-2.39–-2.1) | 150.32(126.37–176.44) | 693.18(601.19–799.5) | 42.84(13.86–81.17) | -1.29(-1.43–-1.14) |
| Norway | 33990.57(29546.83–37703.53) | 326.12(284.23–365.22) | -44.38(-47.79–-41.09) | -3.49(-3.67–-3.31) | 18447.68(15694.84–20611.28) | 303.32(258.94–344.06) | -47.82(-51.24–-44.07) | -3.28(-3.47–-3.1) | 15542.89(13762.83–17205.62) | 349.85(308.14–388.19) | -40.79(-44.41–-36.82) | -3.76(-3.93–-3.6) |
| Oman | 17266.62(14836.39–19889.55) | 1291.27(1116.3–1468.22) | 30.87(7.46–63.34) | -0.16(-0.26–-0.05) | 6041.96(5130.46–7079.5) | 1062.23(895.6–1241.46) | 65.12(29.99–109.42) | 0.47(0.35–0.6) | 11224.66(9229.88–13438.9) | 1515.49(1259.07–1798.76) | 15.29(-11.08–58.74) | -0.59(-0.68–-0.49) |
| Pakistan | 882644.67(743169.48–1132980.22) | 908.84(764.36–1174.74) | 63.57(31.49–111.93) | 0.05(-0.09–0.18) | 456270.58(365721.7–626078.2) | 968.58(777.11–1332.62) | 81.15(42.93–146.27) | 0.15(0.02–0.28) | 426374.09(318775.41–598163.78) | 848.25(633.03–1184.44) | 47.91(13.14–99.97) | -0.08(-0.21–0.06) |
| Palau | 216.5(176.81–264.15) | 1141.16(942.65–1373.36) | 38.47(12.77–71.76) | -0.13(-0.25–-0.01) | 87.25(70.09–106.11) | 963.37(778.59–1149.62) | 44.27(15.73–79.81) | 0(-0.13–0.14) | 129.26(103.77–163.83) | 1316.07(1086.9–1627.21) | 34.7(-0.32–77.49) | -0.23(-0.34–-0.12) |
| Palestine | 31202.38(27202.73–35383.55) | 1590.94(1386.19–1793.74) | 54.61(26.92–87.02) | -0.42(-0.53–-0.32) | 17027.63(14908.87–19308.99) | 1614.44(1413.39–1830.75) | 52.86(25.03–87.38) | -0.41(-0.52–-0.3) | 14174.75(11797.75–16382.95) | 1572.89(1306.66–1802.24) | 56.86(23.93–98.82) | -0.43(-0.53–-0.32) |
| Panama | 14547.42(11651.99–17894.34) | 348.63(278.73–429.64) | 5.97(-10.59–24.73) | -2.03(-2.22–-1.84) | 7072.89(5590.67–8626.05) | 315.34(251.33–384.11) | 5.77(-11.73–25.62) | -2.2(-2.4–-2) | 7474.53(5895.55–9203.69) | 384.94(302.79–475.67) | 6.06(-12.66–27.42) | -1.86(-2.04–-1.67) |
| Papua New Guinea | 27779.18(20223.05–39253.42) | 630.6(469.03–870.24) | 36.96(15.48–63.28) | 0.34(0.18–0.51) | 13306.81(10056.01–17763.47) | 618.27(458.23–829.3) | 41.99(16.36–73.09) | 0.68(0.5–0.85) | 14472.37(9438.28–24532.61) | 641.23(428.65–1069.21) | 32.68(7.12–67.92) | 0.02(-0.14–0.18) |
| Paraguay | 29799.44(23594.16–38241.77) | 574.09(453.67–735.17) | 18.87(-1.15–53.76) | -1.17(-1.33–-1.01) | 13405.5(10535.45–16581.73) | 474.54(372.99–590.55) | 19.27(1.8–41.46) | -1.33(-1.51–-1.16) | 16393.94(12650.23–22082.05) | 685.3(532.41–924.05) | 18.46(-7.84–81.48) | -1.03(-1.18–-0.88) |
| Peru | 77814.44(60798.6–99897.75) | 240.67(187.87–309.5) | 75.78(42.05–127.34) | -2.6(-2.82–-2.37) | 39658.52(30863.91–50233.61) | 233.37(181–296.35) | 80.81(39.36–130.47) | -2.71(-2.94–-2.48) | 38155.92(29231.2–51574.55) | 248.72(190.29–337.65) | 70.68(33.96–146.87) | -2.47(-2.7–-2.25) |
| Philippines | 587348.2(491511.7–680487.6) | 843.51(714.13–967.94) | 175.28(124.72–213.5) | 1.29(1.14–1.44) | 299454.74(242647.53–354250.22) | 797.95(652.99–935.97) | 163.41(130.41–199.3) | 0.91(0.76–1.06) | 287893.46(226488.17–358067.4) | 892.25(709.08–1098.43) | 185.82(100.13–232.54) | 1.67(1.52–1.82) |
| Poland | 530257.78(457390.75–606223.51) | 736.74(635.36–840.05) | 3.58(-3.23–9.81) | -2.38(-2.5–-2.25) | 301403.17(249086.9–355715.93) | 667.71(556.69–788.07) | -4.95(-12.92–1.36) | -2.64(-2.77–-2.51) | 228854.61(186377.46–272858.36) | 804.09(655.34–954.85) | 13.97(6.23–21.01) | -2.04(-2.16–-1.92) |
| Portugal | 141331.39(125693.31–154007.63) | 495.01(442.13–538.13) | -45.75(-50.38–-41.28) | -5.56(-5.69–-5.43) | 80344.07(69205.34–88709.06) | 454(397.01–502.42) | -46.81(-52.29–-41.49) | -5.45(-5.59–-5.31) | 60987.32(54785.98–66828.41) | 547(492.99–597) | -45.46(-50.81–-40.29) | -5.71(-5.83–-5.59) |
| Puerto Rico | 16443.2(13386.58–19799.9) | 215.22(175.37–258.9) | 14.62(3.57–26.24) | -2.47(-2.7–-2.23) | 9337.11(7504.26–11202.05) | 210.67(172.04–251.76) | 10.95(-1.05–24.04) | -2.37(-2.61–-2.13) | 7106.08(5659.63–8624.93) | 220.27(176.02–266.92) | 13.95(-0.46–29.26) | -2.56(-2.79–-2.34) |
| Qatar | 3790.89(2990.01–4599.32) | 577.32(476.3–725) | -0.91(-19.02–16.19) | -1.56(-1.7–-1.41) | 1190.76(958.93–1452.87) | 876.14(716.55–1074.43) | 4.06(-14.85–26.21) | -0.03(-0.16–0.1) | 2600.13(2025.32–3207.15) | 489.85(389.51–641.07) | -1.1(-22.37–22.09) | -2.09(-2.24–-1.93) |
| Republic of Congo | 22095.66(17657.54–27589.05) | 1035.48(829.39–1281.88) | 80.42(52.15–116.68) | -0.65(-0.77–-0.52) | 12778.44(10091.13–16344.04) | 423.64(357.08–486.96) | 82.12(47.84–127.75) | -4.94(-5.08–-4.8) | 9317.22(6967.69–13030.84) | 943.79(710.59–1286.39) | 76.47(38.83–125.42) | -1.13(-1.26–-1) |
| Republic of Korea | 413614.7(360533.95–480454.38) | 482.92(417.58–556.91) | -10.41(-22.39–24.79) | -5.02(-5.15–-4.9) | 213980.49(180545.73–246193.56) | 981.52(872.31–1106.85) | -13.82(-26.85–0.01) | -0.7(-0.82–-0.59) | 199634.21(175098.74–250214.89) | 560.02(491.21–695.5) | -8.05(-23.05–57.63) | -5.28(-5.4–-5.16) |
| Republic of Moldova | 65497.55(58037.51–74259.8) | 1132(1004.38–1280.43) | 57.42(41.16–75.2) | -0.25(-0.36–-0.14) | 34675.77(30813.74–39151.22) | 1256.48(1075.11–1459.9) | 42.9(26.35–60.37) | -2.17(-2.27–-2.07) | 30821.77(26909.79–35009.32) | 1350.89(1184.72–1530.01) | 78.99(56.76–110.11) | 0.26(0.15–0.36) |
| Romania | 551540.85(473100.57–638896.04) | 1386.01(1189.6–1603.94) | 22.41(6.62–35.98) | -2(-2.09–-1.9) | 306950.18(262163.92–356195.86) | 1542.51(1316.92–1777.46) | 20.36(4.7–35.48) | -2.38(-2.47–-2.3) | 244590.67(208831.6–288025.26) | 1546.18(1323.98–1820.32) | 23.82(6.4–42.97) | -1.79(-1.88–-1.7) |
| Russia | 4143600.69(3685649.02–4651105.04) | 1754.56(1561.71–1969.03) | -10.35(-14.88–-5.93) | -2.18(-2.26–-2.1) | 2437724.49(2089705.56–2802048.47) | 647.27(496.88–862.74) | -19(-24.1–-14.89) | -1.63(-1.78–-1.48) | 1705876.2(1426953.13–1999511.58) | 2050.76(1726.85–2395.11) | 4.73(-1.23–12.15) | -2.04(-2.11–-1.97) |
| Rwanda | 32580.17(25495.52–41696.43) | 659.31(513.42–840.67) | 139.22(78.8–207.26) | -1.69(-1.84–-1.54) | 19178.63(14876.01–24965.3) | 1116.13(972.6–1284.61) | 151.96(79.85–231.01) | -2.23(-2.33–-2.12) | 13401.54(9079.47–18356.65) | 676.77(454.45–929.98) | 121.84(54.66–210.32) | -1.74(-1.88–-1.6) |
| Saint Kitts and Nevis | 607.05(527.17–697.8) | 1126.7(992.4–1281.57) | -37.84(-45.03–-30.34) | -2.29(-2.4–-2.18) | 320.53(278.16–369.78) | 746.45(643.45–853.6) | -35.4(-43.72–-24.97) | -3.06(-3.2–-2.93) | 286.53(243.53–330.43) | 1087.62(948.44–1232.6) | -35.57(-46.89–-22.6) | -2.4(-2.51–-2.29) |
| Saint Lucia | 1523.94(1304.74–1750.83) | 743.14(638.71–852.89) | -2.09(-14.63–13.92) | -2.82(-2.96–-2.69) | 853.94(735.51–979.51) | 862.53(756.58–971.95) | -5.16(-15.93–8.81) | -1.6(-1.73–-1.47) | 670(560.93–795.14) | 729.57(616.38–860.08) | 5.96(-12.73–35.32) | -2.49(-2.63–-2.35) |
| Saint Vincent | 971.02(851.92–1095.46) | 763.46(669.83–857.66) | -11.35(-20.62–-0.96) | -1.74(-1.87–-1.6) | 550.35(482.75–621.63) | 862.53(756.58–971.95) | -10.84(-20.62–-0.48) | -1.6(-1.73–-1.47) | 420.67(362.35–482.12) | 659.2(568.09–749.57) | -7.27(-21.59–9.12) | -1.81(-1.95–-1.66) |
| Samoa | 1208.37(993.91–1442.69) | 888.03(736.07–1055.61) | 27.86(7.77–50.23) | -0.42(-0.56–-0.29) | 644.13(520.08–793.73) | 904.94(732.46–1113.62) | 32.82(11.72–60.38) | 0.09(-0.05–0.23) | 564.24(445.51–694.67) | 867.36(690.14–1053.45) | 20.98(-4.82–53.25) | -0.96(-1.09–-0.83) |
| San Marino | 267.96(200.12–346.48) | 359.6(269.18–464.62) | -6.65(-18.99–7.98) | -1.6(-1.8–-1.41) | 141.41(104.62–185.98) | 326.97(243.02–431.46) | -9.88(-24.39–7.68) | -1.34(-1.55–-1.13) | 126.55(92.45–164.89) | 396.24(287.79–516.9) | -3.65(-20.16–15.57) | -1.92(-2.1–-1.73) |
| Sao Tome and Principe | 915.4(752.98–1140.04) | 935.21(773.83–1163.82) | 127.47(86.98–183.92) | 0.2(0.07–0.34) | 537.11(423.72–713.09) | 1048.83(827.75–1401.81) | 113.3(71.27–176.84) | 0.04(-0.09–0.17) | 378.29(298.03–470.15) | 805.84(640.64–988.37) | 150.71(91.35–224.55) | 0.42(0.27–0.57) |
| Saudi Arabia | 207936.05(160132.18–249404.78) | 1300.77(1026.88–1516.11) | 64.2(28.75–103.23) | -0.66(-0.77–-0.56) | 88551.17(70719.48–108458.29) | 1364.02(1110.24–1642.35) | 70.41(29.42–124.53) | -0.71(-0.81–-0.6) | 119384.88(87472.01–145143.37) | 1251(913.29–1469.63) | 61.72(21.83–105.13) | -0.63(-0.74–-0.53) |
| Senegal | 46473.77(38404.3–55708.7) | 657.46(546.96–788.12) | 105.66(65.1–151.7) | -0.65(-0.8–-0.5) | 26364.81(21631.79–31358.84) | 713.23(590.78–851.02) | 115.23(73.55–164.8) | -0.6(-0.75–-0.45) | 20108.95(15844.36–25772.61) | 595.68(470.34–752.87) | 93.41(44.38–152.73) | -0.74(-0.9–-0.59) |
| Serbia | 313708.61(265680.9–370741.43) | 1970.97(1674.85–2323.13) | 24.33(7.14–39.25) | -1.33(-1.41–-1.24) | 177027.34(149718.88–208395.41) | 1962.57(1670.6–2304.61) | 20.46(7.06–35.78) | -1.5(-1.58–-1.42) | 136681.28(112727.34–164454.16) | 1954.38(1626.23–2345.9) | 26.77(-0.8–47.6) | -1.09(-1.17–-1.01) |
| Seychelles | 775.67(656.53–912.94) | 775.54(661.03–905.17) | -11.59(-20.23–-1.26) | -1.06(-1.2–-0.92) | 371.48(304.34–439.87) | 681.31(560.1–807.73) | -4.84(-14.92–6.45) | -0.51(-0.67–-0.36) | 404.19(324.43–494.05) | 866.88(700.93–1050.02) | -16.62(-29.1–-0.39) | -1.73(-1.86–-1.6) |
| Sierra Leone | 28704.66(22331.9–37072.18) | 796.96(629.24–1004.15) | 54.71(29.99–86.51) | -0.24(-0.39–-0.1) | 16199.27(12519.12–20717.25) | 872.54(687.23–1096.9) | 66.02(33.22–110.19) | -0.01(-0.14–0.13) | 12505.39(8904.63–17297.61) | 716.42(507.92–979.35) | 41.89(10.75–80.05) | -0.56(-0.7–-0.41) |
| Singapore | 20596.1(17455.92–23673.12) | 282.11(239.46–323.58) | -38.52(-43.56–-33.68) | -5.02(-5.19–-4.85) | 11601.53(9616.85–13455.55) | 292.71(242.94–341.28) | -38.68(-44.45–-33.33) | -4.85(-5.02–-4.68) | 8994.57(7683.8–10316.53) | 266.73(230.56–301.79) | -38.97(-44.57–-32.93) | -5.23(-5.4–-5.06) |
| Slovakia | 79047.89(65498.97–94039.77) | 861.8(714.93–1021.93) | 2.16(-6.57–12.57) | -1.68(-1.81–-1.56) | 42341.83(34885.76–50193.17) | 759.66(628.38–899.48) | -6.89(-15.72–4.4) | -1.94(-2.07–-1.81) | 36706.06(29704.58–44596.63) | 986.72(803.55–1191.3) | 10.61(-3–24.62) | -1.39(-1.5–-1.27) |
| Slovenia | 21240.22(17202.18–26157.34) | 446.16(362.15–545.57) | -38.02(-45.14–-31.62) | -4.54(-4.69–-4.39) | 11710.08(9338.56–14838.68) | 395.52(316.34–488.02) | -39.94(-47.37–-32.97) | -4.58(-4.74–-4.42) | 9530.15(7701.73–11685.44) | 517.48(420.61–636) | -35.62(-44.54–-27.84) | -4.54(-4.68–-4.41) |
| Solomon Islands | 3721.56(2973.67–4696.72) | 1406.71(1153.39–1747.2) | 55.38(27.64–81.52) | 0.44(0.32–0.55) | 1953.13(1573.81–2516.01) | 1551.93(1270.25–1928) | 76.75(42.7–116.13) | 0.6(0.49–0.71) | 1768.43(1181.14–2619.67) | 1269.02(910.2–1819.13) | 36.88(5.37–68.87) | 0.21(0.09–0.33) |
| Somalia | 50034.88(36887.75–68046.04) | 880.39(645.49–1203.65) | 69.98(34–115.55) | 0.21(0.07–0.35) | 29446.21(21272.05–41216.3) | 884.56(634.72–1248.74) | 83.46(44.02–129.8) | 0.2(0.06–0.35) | 20588.66(13957.13–29321.74) | 869.22(596.3–1256.89) | 53.9(9.35–130.71) | 0.18(0.04–0.33) |
| South Africa | 329002.23(297573.68–356567.35) | 832.17(755.61–898.59) | 47.64(31.85–66.05) | 0.31(0.17–0.44) | 194649.21(173483.34–213910.14) | 815.25(726.34–894.06) | 45.41(29.12–64.55) | 0.39(0.26–0.53) | 134353.02(120380.82–146722.62) | 841.21(758.37–912.85) | 47.51(26.03–72.67) | 0.11(-0.02–0.24) |
| South Sudan | 23216.45(16878.13–32904.11) | 692.84(492.83–983.59) | 51.64(17.59–100.54) | -0.26(-0.42–-0.11) | 11857.16(8960.4–15103.28) | 727.59(534.8–947.64) | 48.72(15.39–87.77) | -0.34(-0.49–-0.19) | 11359.29(7668.16–18883.62) | 659.59(439.18–1088.89) | 54.04(7.05–132.44) | -0.15(-0.31–0.01) |
| Spain | 290082.76(252227.36–321866.33) | 254.22(223.83–282.58) | -45.11(-49.55–-40.33) | -4.67(-4.87–-4.48) | 172449.73(146572.5–194509.93) | 243.56(208.64–274.64) | -47.6(-52.5–-42.51) | -4.61(-4.82–-4.41) | 117633.03(104942.34–130540.62) | 264.14(235.2–293.29) | -43.61(-48.97–-37.57) | -4.77(-4.96–-4.59) |
| Sri Lanka | 177959.98(140903.13–223337.33) | 780.18(616.53–971) | 65.99(38.78–91.56) | -0.99(-1.12–-0.86) | 90652.89(71620.06–112021.75) | 701.44(553.79–867.27) | 71.09(44.27–110.18) | -1.06(-1.19–-0.92) | 87307.08(66349.62–111910.39) | 879.99(677.01–1112) | 58.95(27.69–92.27) | -0.83(-0.95–-0.7) |
| Sudan | 304172.07(232831.72–427019.77) | 1692.75(1303.49–2360.63) | 202.11(133.68–282.2) | 0.45(0.35–0.55) | 153815.57(113326.05–204131.95) | 1840.49(1357.95–2416.34) | 196.97(129.9–279.41) | 0.57(0.47–0.67) | 150356.49(103645.76–252869.25) | 1565.88(1096.84–2623.12) | 207.73(120.13–316.35) | 0.36(0.25–0.46) |
| Suriname | 4475.09(3758.04–5201.88) | 796.45(672.11–922.15) | 50.19(32.74–69.29) | -0.73(-0.87–-0.59) | 2365.48(2003.85–2783.67) | 758.86(643.35–892.14) | 44.05(23.78–67.3) | -0.86(-1–-0.72) | 2109.61(1723.04–2534.31) | 833.44(685.07–998.71) | 56.79(33.92–83.32) | -0.63(-0.76–-0.49) |
| Sweden | 81892.05(72485.12–90905.77) | 358.85(312.82–402.85) | -25.84(-31.05–-19.68) | -2.48(-2.66–-2.3) | 43832.64(37683.07–49280.23) | 338.67(291.87–386.76) | -28.48(-33.77–-21.54) | -2.18(-2.37–-1.98) | 38059.41(33923.57–42306.34) | 379.84(334.84–424.82) | -23.13(-29.11–-16.59) | -2.84(-3.01–-2.66) |
| Switzerland | 44320.07(38033.61–50139.54) | 221.75(192.24–250.98) | -39.58(-45.14–-33.33) | -3.73(-3.95–-3.51) | 25435.62(21226.43–29139.01) | 212.88(180.6–244.59) | -42.21(-48.34–-35.11) | -3.38(-3.61–-3.15) | 18884.45(16687.72–21058) | 230.88(204.72–257.11) | -37.3(-43.2–-30.7) | -4.21(-4.42–-4) |
| Syrian Arab Republic | 116536.89(92554.13–145609.21) | 1069.91(858.84–1325.06) | 74.31(43.33–113.59) | -1.58(-1.69–-1.46) | 57396.06(46538.81–70328.97) | 1177.89(965.19–1417.42) | 76.09(41.32–117.81) | -1.5(-1.61–-1.39) | 59140.84(45367.57–76344.76) | 1016.53(794.7–1305.22) | 72.27(33.45–127.52) | -1.49(-1.61–-1.37) |
| Taiwan (Province of China) | 155428.88(128706.76–183906.9) | 402.36(332.66–475.89) | -17.22(-24.67–-9.83) | -3.54(-3.7–-3.37) | 82093.59(66947.07–97895.23) | 395.59(319.1–472.91) | -14.83(-23.61–-6.21) | -3.69(-3.86–-3.53) | 73335.29(60654.6–88344.81) | 410.96(340.18–492.35) | -20.82(-28.81–-12.52) | -3.27(-3.44–-3.1) |
| Tajikistan | 24033.08(19642.43–30096.57) | 660.61(547.69–805.16) | 120.63(81.45–174.65) | 1.96(1.79–2.13) | 12737.18(10488.39–15097.61) | 651.32(540.75–780.09) | 117.71(77.01–163.92) | 1.94(1.77–2.11) | 11295.9(8536.75–16203.11) | 679.45(523.45–914.43) | 126.02(71.82–201.39) | 2(1.83–2.17) |
| Thailand | 464144.69(368546.4–572097.59) | 471.48(376.25–579.94) | 73.72(47.44–100.54) | -1.75(-1.92–-1.58) | 235062.59(189024.88–289054.27) | 433.59(349.15–531.6) | 74.82(48.53–106.61) | -1.58(-1.76–-1.41) | 229082.1(177608.95–291472.58) | 512.89(400.47–649.62) | 71.45(36.57–109.41) | -1.94(-2.11–-1.78) |
| Timor-Leste | 9292.59(6993.6–12803.83) | 1280.07(976.54–1756.59) | 546.24(402.96–711.42) | 1.23(1.1–1.35) | 4310.14(3568.23–5150.98) | 1196.63(999.57–1422.32) | 512.33(397.88–653.08) | 0.81(0.68–0.94) | 4982.45(3114.32–8201.78) | 1367.34(871.81–2220.24) | 582.18(394.54–816.22) | 1.65(1.52–1.78) |
| Tobago | 12295.5(9760.87–15439.98) | 697.88(555.44–874.09) | -4.24(-16.04–10.1) | -2.68(-2.82–-2.55) | 5806.14(4613.51–7243.17) | 714.71(588.69–872.22) | -0.45(-14.79–16.61) | -0.77(-0.92–-0.62) | 6489.36(4995.52–8318.86) | 786.24(608.57–1001.89) | -7.37(-20.8–10.84) | -2.77(-2.9–-2.65) |
| Togo | 24927.14(20381.22–30260.68) | 756.42(628.14–909.15) | 102.5(70.39–144.96) | -0.5(-0.64–-0.35) | 13563.62(10998.23–16456.59) | 979.14(791.22–1210.69) | 104.5(69.9–145.87) | -0.09(-0.22–0.04) | 11363.52(8689.26–14543.98) | 811.55(638.62–1030.64) | 100.69(55.18–156.78) | -0.15(-0.29–-0.01) |
| Tokelau | 10.13(8.51–12.22) | 815.94(682.56–980.47) | 26.62(6.95–52.8) | -0.34(-0.47–-0.2) | 6.08(4.92–7.49) | 629.86(516.93–755.61) | 30.41(8.13–61.84) | 0.14(-0.03–0.31) | 4.05(3.36–4.91) | 647.67(537.66–780.08) | 22.22(-6.42–58.2) | -0.63(-0.79–-0.48) |
| Tonga | 489.05(409.82–574.07) | 626.99(527.76–735.92) | 45.46(24.85–73.65) | 0(-0.16–0.17) | 275.73(226.44–330.71) | 614.42(488.5–765.25) | 58.56(34.51–89.88) | -2.6(-2.75–-2.46) | 213.31(173.92–261.78) | 614.95(501.01–752.38) | 31.55(6.34–65.34) | -0.17(-0.33–-0.01) |
| Trinidad | 12295.5(9760.87–15439.98) | 697.88(555.44–874.09) | -4.24(-16.04–10.1) | -2.68(-2.82–-2.55) | 5806.14(4613.51–7243.17) | 614.42(488.5–765.25) | -0.45(-14.79–16.61) | -2.6(-2.75–-2.46) | 6489.36(4995.52–8318.86) | 786.24(608.57–1001.89) | -7.37(-20.8–10.84) | -2.77(-2.9–-2.65) |
| Tunisia | 128085.96(98884.56–163157.52) | 1090.45(848.39–1382.72) | 159.27(110.91–207.34) | 0.16(0.04–0.28) | 64674.31(49913.23–80795.59) | 1056.58(818.57–1321.43) | 164.09(110.5–223.04) | 0.07(-0.05–0.2) | 63411.65(46796.41–83312.75) | 1127.29(836.4–1473.77) | 153.95(94.99–218.42) | 0.25(0.13–0.37) |
| Turkey | 551063.79(459966.94–649248.3) | 662.68(552.88–779.5) | 179.76(105.66–237.04) | 0.39(0.23–0.55) | 305046.73(254643.69–361113.56) | 674.58(562.72–797.68) | 167.75(110.93–233.27) | 0.25(0.1–0.41) | 246017.06(202488.71–296238.12) | 644.19(530.24–774.77) | 189.85(87.84–271.48) | 0.54(0.38–0.7) |
| Turkmenistan | 60520.24(48085.02–74942.07) | 1671.89(1342.24–2048.76) | 154.95(112.59–206.55) | 0.79(0.69–0.9) | 29131.01(22679.01–36524.66) | 1458.51(1146.1–1818.84) | 129.38(84.79–185.34) | 0.46(0.35–0.57) | 31389.23(24248.4–39443.58) | 1946.75(1528.52–2435.06) | 189.51(138.51–283.71) | 1.11(1.01–1.2) |
| Tuvalu | 92.92(74.92–113.96) | 992.71(806.07–1213.72) | 74.62(41.46–114.12) | -0.27(-0.39–-0.14) | 51.39(40.51–63.33) | 1031.66(814.54–1258.45) | 92.55(54.77–140.67) | 0.13(0–0.26) | 41.53(32.94–52.21) | 942.67(758.09–1174.53) | 58.18(16.75–113.56) | -0.8(-0.93–-0.67) |
| Uganda | 89401.74(71121.4–107724.66) | 713.17(564.55–857.55) | 152.97(104.27–207.78) | -0.33(-0.48–-0.18) | 51560.93(40059.07–64628.85) | 702.15(537.89–883.48) | 184.13(125.72–255.94) | -0.36(-0.51–-0.21) | 37840.81(28719.06–50156.13) | 722.74(543.41–948.39) | 121.99(74.68–182.88) | -0.26(-0.4–-0.11) |
| UK | 431116.06(386414.17–464260.99) | 310.2(278.06–336.58) | -46.38(-49.45–-43.88) | -3.97(-4.15–-3.79) | 241670.24(211012.35–263251.7) | 1354.17(1128.18–1611.51) | -48.63(-51.89–-45.34) | -2.35(-2.45–-2.26) | 189445.81(174188.02–202752.25) | 320.38(293.14–343.91) | -43.32(-46.17–-40.95) | -4.21(-4.38–-4.03) |
| Ukraine | 1255383.79(1097926.02–1431910.6) | 1635.07(1431.43–1864.27) | -21.26(-28.1–-13.5) | -1.98(-2.07–-1.89) | 688264.33(572697.87–817477.01) | 1466.53(1184.15–1829.91) | -25.18(-32.13–-18.16) | -0.89(-0.97–-0.81) | 567119.45(469061.27–680875.62) | 2045.14(1695.66–2434.07) | -10.78(-20.89–0.98) | -1.62(-1.71–-1.54) |
| United Arab Emirates | 54428.2(41442.81–69792.18) | 1415.24(1128.21–1749.32) | 46.6(12.66–90.78) | -1.4(-1.49–-1.31) | 14691.79(11578.83–18309.18) | 298.55(262.66–329.21) | 56.19(22.75–96.27) | -3.78(-3.97–-3.6) | 39736.41(29091.9–52848.87) | 1398.02(1078.5–1730.24) | 46.26(6.59–109.51) | -1.63(-1.72–-1.53) |
| United Republic of Tanzania | 196258.41(153058.64–260306.71) | 881.5(695.66–1143.22) | 187.05(119.37–283.3) | 1.03(0.88–1.17) | 102966.28(81951.75–124805.81) | 853.23(678.66–1032.88) | 189.85(131.07–255.15) | 1.11(0.96–1.26) | 93292.13(65248.24–144667.03) | 910.47(642.85–1380.84) | 184.36(78.44–369.45) | 0.93(0.78–1.07) |
| Uruguay | 33893.9(30324.66–37006.4) | 485.71(422.83–544.23) | -26.06(-32.3–-19.15) | -2.62(-2.76–-2.47) | 19801.39(17304.12–21768.97) | 369.4(315.95–421.54) | -25.01(-32.15–-17.19) | -1.61(-1.81–-1.42) | 14092.5(12746.45–15575.76) | 615.55(558–680.94) | -29.15(-36.49–-20.22) | -2.63(-2.77–-2.5) |
| USA | 2056104.53(1787995.27–2311781.36) | 570.35(512.63–623.3) | -18.01(-21.46–-14.02) | -1.74(-1.93–-1.54) | 1208307.24(1040903.06–1367165.9) | 421.26(359.18–482.43) | -21.88(-25.48–-18.1) | -0.9(-1.09–-0.71) | 847797.29(738635.36–955035.59) | 344.99(298.84–390) | -13(-18.35–-7.69) | -1.9(-2.1–-1.7) |
| Uzbekistan | 289083.66(246588.91–338016.95) | 360.1(310.57–408.06) | 45.33(15.5–73) | 0.1(0.01–0.19) | 133948.55(114443.9–156862.09) | 529.2(467.57–582.49) | 30.32(-2.25–58.44) | -2.58(-2.73–-2.43) | 155135.11(129639.47–183644.18) | 1996.83(1710.33–2314.03) | 61.56(31.72–99.25) | 0.18(0.09–0.26) |
| Vanuatu | 2018.69(1600.22–2638.85) | 1704.81(1478.48–1957.03) | 54.77(33.15–80.35) | -0.13(-0.24–-0.01) | 966.66(739.52–1254.65) | 1480.29(1282.73–1704.83) | 67.18(41.3–100.12) | -0.03(-0.13–0.07) | 1052.04(802.54–1438.99) | 1237.68(949.18–1716.71) | 44.77(19.25–74.38) | -0.26(-0.38–-0.15) |
| Venezuela (Bolivarian Republic of) | 109806.88(88336.67–136178.97) | 1245.13(996.99–1640.11) | 45.06(20.35–70.95) | -1.24(-1.44–-1.04) | 54912.81(44350.49–67216.83) | 1253.16(965.46–1631.16) | 40.27(14.94–67) | 0.04(-0.08–0.16) | 54894.07(42853.95–69470.39) | 453.78(354.58–570.05) | 50.71(17.88–83.83) | -1.07(-1.26–-0.89) |
| Viet Nam | 1163673.15(948468.4–1373609.91) | 409.38(328.77–506.6) | 108.84(60.01–162.77) | 0.69(0.58–0.8) | 496825.07(403831.47–604326.1) | 370.93(299.34–455.21) | 96.73(59.08–161.46) | -1.42(-1.62–-1.21) | 666848.08(510889.81–821360.65) | 2008.9(1564.94–2427.68) | 119.81(57.16–181.61) | 0.92(0.83–1.02) |
| Virgin Islands | 861.64(753.57–970.34) | 1433.65(1172.72–1675.18) | 64.8(41.34–88.37) | -0.12(-0.3–0.07) | 419.38(355.01–483.34) | 1035.5(841.99–1263.49) | 48.6(25.96–74.78) | 0.39(0.26–0.52) | 442.25(370.4–521.34) | 560.09(474.79–646.83) | 88.23(49.12–131.57) | 0.76(0.57–0.94) |
| Yemen | 212194.83(168265.06–276241.1) | 1716.66(1370.21–2197.14) | 219.77(153.5–301.25) | 0.74(0.63–0.84) | 113472.99(88582.68–147747.26) | 1793.92(1416.12–2340.8) | 226.59(158.95–314.06) | 0.97(0.87–1.07) | 98721.84(75988.81–132851.89) | 1635.75(1267.72–2173.51) | 213.56(130.76–321.92) | 0.51(0.4–0.61) |
| Zambia | 59119.02(47798.6–76135.99) | 1047.99(851.44–1346.69) | 211.84(136.52–292.55) | 1.12(0.98–1.26) | 33333.9(27116.46–41899.17) | 1088.21(881.27–1354.94) | 235.24(157.29–335.01) | 0.75(0.61–0.88) | 25785.13(18260.93–39510.39) | 993.27(706.22–1475.36) | 191.78(104–290.14) | 1.44(1.29–1.59) |
| Zimbabwe | 62634.84(50528.38–77416.65) | 1052.71(850.03–1298.32) | 43.35(19.92–72.95) | 0.99(0.86–1.12) | 39090.66(30148.28–49967.03) | 1104.69(853.48–1409.31) | 52.4(21.28–93.56) | 1.47(1.34–1.61) | 23544.18(19044.84–32519.62) | 969.67(784.45–1349.94) | 28.9(1.57–62.62) | 0.34(0.21–0.47) |

Abbreviations: DALYs, disability-adjusted life years; ASDR, age-standardized DALYs rate; EAPC, estimated annual percentage change;
